# Clinical similarity, economic divergence: cost-effectiveness and population health implications of dietary, pharmacological, and exercise interventions in older adults with type 2 diabetes: a secondary analysis of a randomized controlled trial

**DOI:** 10.1186/s12877-026-07994-7

**Published:** 2026-07-25

**Authors:** Limor Dina Gonen, Assaf Buch, Roy Eldor, Iris Yaish, Sharon Barak

**Affiliations:** 1 Department of Economics and Business Administration, Ariel University, P.O.B. 3, Kiryat HaMada, Ariel, 40700 Israel; 2 Department of Nutritional Sciences, School of Health Sciences, Ariel University, Ariel, Israel; 3 Metabolism and Hypertension, Tel Aviv Sourasky Medical Center, Institute of Endocrinology, Tel Aviv, Israel; 4 The Faculty of Medicine, Tel Aviv University, Tel Aviv, Israel; 5 Department of Nursing, School of Health Sciences, Ariel University, Ariel, 40700 Israel

**Keywords:** Ageing, Frailty, Cost-effectiveness, Health economics, Lifestyle intervention, Population health, Preventive care, Healthy ageing, Social determinants of health, Health equity

## Abstract

**Background:**

Population ageing is increasing the burden of chronic disease and frailty, raising important questions regarding how health systems define and allocate value. While pharmacological treatments dominate clinical care, lifestyle-based interventions may offer broader benefits for healthy ageing. However, integrated clinical and economic evidence in older populations remains limited.

**Methods:**

We conducted a secondary analysis of the CEV-65 randomized controlled trial (*n* = 100 older adults aged 65 years and older with type 2 diabetes), comparing a Mediterranean diet (VegMedD), pharmacological treatment (empagliflozin), and exercise (circuit resistance training). Outcomes included changes in glycemic control (HbA1c) and frailty, alongside a composite responder measure. Economic evaluation included trial-based cost-effectiveness analyses (using the composite responder outcome based on improvement in both HbA1c and frailty) and cost–utility analyses using quality-adjusted life years (QALYs) derived from SF-12 data via a validated mapping approach. Adjusted regression models, propensity score weighting, and probabilistic sensitivity analyses were applied. Scenario analyses assessed sensitivity to pharmacological pricing assumptions.

**Results:**

All interventions improved glycemic control, with no statistically significant between-group differences. Frailty improvements were modest. Pharmacological treatment achieved the highest responder rate, whereas non-pharmacological interventions were substantially less costly. In cost-effectiveness analysis, lifestyle interventions were associated with lower costs but reduced responder-based effectiveness. In contrast, cost–utility analysis indicated that both dietary and exercise interventions were economically dominant, yielding lower costs and higher QALYs under current pricing conditions. The dietary intervention demonstrated the highest probability of cost-effectiveness across conventional willingness-to-pay thresholds. Scenario analyses showed that responder-based conclusions were sensitive to pharmacological pricing, whereas cost–utility findings remained comparatively robust.

**Conclusions:**

Interventions with similar short-term clinical outcomes may differ substantially in their economic and population health value. Within the short-term trial horizon, lifestyle-based strategies were associated with more favorable cost–utility profiles. These findings should be interpreted as exploratory and should not be extrapolated to long-term comparative effectiveness, particularly given the established long-term cardiovascular and renal benefits of SGLT2 inhibitors that could not be captured within the 10-week follow-up period.

**Clinical trial number:**

ClinicalTrials.gov (Identifier: NCT03560375; registered on June 5, 2018) https://clinicaltrials.gov/study/NCT03560375?tab=history.

**Supplementary Information:**

The online version contains supplementary material available at https://doi.org/10.1186/s12877-026-07994-7.

## Highlights

Dietary and exercise-based interventions reduced costs relative to pharmacological treatment.

Short-term clinical improvements were modest and broadly similar across groups.

The dietary intervention showed the highest probability of cost-effectiveness.

Non-pharmacological strategies yielded slightly higher short-term QALYs than pharmacological treatment, with long-term implications remaining uncertain.

Findings support preventive, community-based ageing policies and broader value frameworks beyond clinical endpoints alone.

**Supplementary Information:**

The online version contains supplementary material available at https://doi.org/10.1186/s12877-026-07994-7.

## Introduction

Population ageing is reshaping the epidemiological and economic landscape of health systems worldwide, raising fundamental questions about how value in healthcare should be defined and operationalized. As the burden of chronic disease and functional decline increases, the challenge facing policymakers is no longer limited to improving disease-specific outcomes but extends to optimizing quality of life, functional ability, and long-term system sustainability.

As longevity increases, health systems face a growing burden of chronic disease, functional decline, and long-term care needs, with major implications for prevention, service delivery, and resource allocation [1, 2]. Within this broader context, frailty and metabolic dysregulation have emerged as central and interrelated challenges in older populations.

Frailty is widely understood as a state of heightened vulnerability resulting from age-related declines across multiple physiological systems, which reduce resilience to internal and external stressors [3]. It is strongly associated with adverse outcomes, including falls, hospitalization, disability, institutionalization, and mortality [4, 5]. Beyond its clinical significance, frailty has become a major public health concern because of its contribution to healthcare utilization, rising care complexity, and long-term system burden [6]. Environmental conditions may further intensify these risks, as shown by evidence linking environmental hazards to higher fall risk among older adults in long-term care settings [7]. Importantly, growing evidence suggests that frailty is also socially patterned, reflecting cumulative exposures to disadvantage across the life course, including inequalities in nutrition, housing, access to care, and opportunities for physical activity [8, 9].

At the same time, older adults with type 2 diabetes remain highly prevalent in ageing populations. Diabetes in older adults presents distinctive clinical and management challenges, including multimorbidity, functional decline, and competing therapeutic priorities [10]. In older adults, treatment goals extend beyond glycemic control to include preservation of functional ability, reduction of treatment burden, and maintenance of quality of life, making broader evaluative frameworks especially relevant. Importantly, frailty and type 2 diabetes are not separate phenomena. Rather, they often coexist and interact, with impaired metabolic regulation contributing to functional deterioration and frailty amplifying the vulnerability associated with chronic disease. This combination creates a particularly important target for interventions aimed at improving both clinical outcomes and healthy ageing trajectories.

Pharmacological interventions play a central role in the management of diabetes. In particular, empagliflozin has demonstrated important benefits for patients with type 2 diabetes, including reductions in cardiovascular events and mortality [11]. However, although such pharmacological strategies may be clinically effective, they are also associated with direct treatment costs and may not sufficiently address broader functional outcomes or the multidimensional processes that shape vulnerability in later life. Moreover, the dominance of pharmacological approaches in routine care may reflect institutional and reimbursement structures as much as comparative societal value, particularly when broader outcomes such as functioning, independence, and quality of life are considered. In addition, short-term treatment experience may differ across interventions, with pharmacological therapies potentially associated with symptoms that influence perceived well-being, particularly in older adults [12]. Importantly, SGLT2 inhibitors, including empagliflozin, have well-established cardiovascular and renal protective effects, extending beyond glycemic control and including reductions in heart failure and progression of kidney disease across a broad range of patient risk profiles [11, 13, 14]. These clinically meaningful benefits are not fully captured within the short-term time horizon of the present analysis.

Furthermore, commonly used endpoints such as hemoglobin A1c (HbA1c) may be more responsive to dietary and pharmacological interventions, whereas short-duration pragmatic exercise interventions may exert benefits in domains not fully captured by these endpoints. In parallel, non-pharmacological interventions have attracted growing attention as potentially scalable and sustainable approaches for improving health in older adults. Mediterranean dietary patterns have shown substantial benefits for cardiovascular prevention and metabolic health [15], while exercise-based interventions have been increasingly recognized as important tools for preventing and managing frailty and fragility-related decline [16]. From a healthy ageing perspective, such interventions are especially attractive because they may influence both metabolic risk and functional capacity, thereby addressing multiple determinants of health simultaneously [17]. They may also generate benefits that extend beyond disease control, including improved self-management, participation, and perceived well-being (e.g., Wu et al. [18]), all of which are highly relevant in ageing populations [19].

From a policy and health systems perspective, the key question is not only whether interventions are clinically beneficial, but also whether they provide good value for money. Health economic evaluation offers a structured framework for comparing alternative strategies in terms of costs and consequences, including cost-effectiveness and cost-utility analyses [20, 21]. This perspective is particularly important in ageing populations, where interventions may produce modest short-term clinical changes yet still generate meaningful gains in quality of life, independence, and long-term system sustainability. Indeed, a major challenge for ageing policy is that interventions judged similar by conventional biomedical endpoints may nonetheless differ substantially in their consequences for functioning, autonomy, equity, and resource allocation.

Previous economic evaluations have reached varying conclusions regarding the relative value of pharmacological and lifestyle-based interventions. In particular, evidence from the Diabetes Prevention Program (DPP) has shown that pharmacological treatment with metformin may be cost-effective or even cost-saving over extended time horizons [22]. However, such findings are context-dependent and influenced by population characteristics, intervention intensity, and analytic time horizon. As such, their applicability to older populations with established disease, as well as to short-term trial-based evaluations, may be limited. This variability highlights a critical gap in the literature: the extent to which conclusions regarding intervention value depend on the evaluative framework, time horizon, and outcome definition applied.

Despite the growing importance of this issue, several gaps remain in the literature. First, much of the existing evidence addresses either clinical effectiveness or economic value, rather than integrating both within a unified empirical framework. Second, direct comparisons between pharmacological and non-pharmacological interventions in older adults and those with type 2 diabetes in particular remain limited, especially when both metabolic and frailty-related outcomes are considered simultaneously. Third, although healthy ageing frameworks emphasize prevention, function, and person-centered care [1, 2], there remains relatively little trial-based economic evidence comparing dietary, exercise-based, and pharmacological strategies within the same older population. Fourth, existing evaluations often privilege average clinical effects while paying less attention to broader population health implications, including the ways in which social and structural determinants may shape the uptake, sustainability, and distribution of intervention benefits [23, 24].

The present study addresses these gaps by providing an integrated clinical and economic evaluation of dietary, pharmacological, and exercise-based interventions among older adults with type 2 diabetes, all of whom were aged 65 years or older at enrollment, using data from a randomized controlled trial (the CEV-65 study) [25]. Rather than evaluating interventions solely in terms of clinical effectiveness, the study explicitly examines how alternative evaluative frameworks may lead to different conclusions regarding intervention value.

Specifically, the study aims to: (1) compare the effects of these interventions on glycemic control (HbA1c) and frailty-related outcomes; (2) evaluate their relative cost-effectiveness using a trial-based composite responder outcome based on improvement in both HbA1c and frailty; (3) estimate cost–utility using quality-adjusted life years (QALYs); and (4) assess the robustness of findings through multivariable adjustment, mixed-effects modeling, propensity score weighting, and uncertainty analyses.

By integrating clinical, economic, and population health perspectives within a single empirical framework, the study provides a structured test of whether interventions that appear clinically similar may nonetheless diverge in their broader economic and societal value. In doing so, it contributes to ongoing debates on how value should be conceptualized and measured in ageing populations, where policy priorities extend beyond disease control to include functioning, well-being, and long-term system sustainability. Because the present analysis was restricted to the randomized 10-week intervention period, the economic evaluation was designed as a short-term exploratory within-trial assessment rather than a comprehensive long-term economic evaluation.

## Methods

### Study design and data source

This study was conducted as a secondary analysis of a previously registered randomized controlled trial (CEV-65; ClinicalTrials.gov Identifier: NCT03560375; registered on June 5, 2018, prior to or at the initiation of participant recruitment) [25], a multi-arm controlled intervention study designed to examine metabolic, functional, and economic outcomes associated with dietary (VegMedD), pharmacological (empagliflozin), and exercise-based (circuit resistance training) interventions in older adults with type 2 diabetes. Given the aims of the present analysis, the study was framed not only as a comparative effectiveness exercise but also as an assessment of whether different intervention modalities generate different forms of value for ageing populations. Importantly, the present analysis is based on primary individual-level data from the CEV-65 trial [25] rather than on modeled or simulated cohorts, allowing direct comparison of clinical and economic outcomes across intervention arms within the same older population.

Intervention group membership was defined using the Group variable (1 = VegMedD, 2 = Empagliflozin, 3 = Exercise). Participants without valid group assignment were excluded.

The present analysis was restricted to the initial 10-week intervention phase to enable a direct head-to-head comparison of intervention-specific effects prior to the combined intervention phase. This analytical choice allows the isolation of intervention-specific value but may underestimate longer-term or synergistic effects. Accordingly, all economic analyses should be interpreted as exploratory within-trial evaluations of short-term value and were not intended to estimate the long-term economic value of the evaluated interventions. Because the present study is a secondary analysis of a previously completed randomized controlled trial, reporting follows the CONSORT 2010 Statement where applicable to secondary analyses. Details relating to trial conduct (including participant flow, randomization, allocation concealment, and blinding) are reported in the original trial publication. A completed CONSORT checklist and CONSORT flow diagram are provided as supplementary files.

### Study population

The analytic cohort included 100 participants (33 VegMedD, 33 Empagliflozin, 34 Exercise). Eligible participants were adults aged 65 years and older with type 2 diabetes. Baseline analyses were conducted on all participants with available data, whereas outcome-specific analyses used complete cases for each relevant outcome, consistent with a within-trial analytic framework. Because the study addresses questions of broader policy relevance, complete-case analyses were complemented by robustness checks intended to reduce concerns that findings were driven by measured baseline differences between intervention groups.

Given the modest size of the trial, the economic analyses were interpreted with particular attention to uncertainty, especially where responder counts were low in specific arms.

### Outcome measures

The primary metabolic outcome was glycemic control assessed using HbA1c. Change in HbA1c was defined as:

$$\:\varDelta\:HbA1c={HbA1c}_{W10}-{HbA1c}_{W0}$$

Negative values indicate improvement.

The original and modified frailty phenotype measures have a strong correlation (*r* = 0.89; *P* < 0.000) and agreement (κ = 0.84; 95% confidence interval = 0.81-0.86) [26]. In the present study, the modified Fried phenotype was used because the available trial data did not permit differentiation between intentional and unintentional weight loss, and because the available physical activity measure did not correspond to the original Fried operationalization while also representing a direct intervention target. Accordingly, frailty was operationalized using the available trial variables corresponding to exhaustion, functional status (self-report of limitations in walking half a mile), grip strength, and gait speed, while excluding the weight-loss and physical-activity components. This approach was intended to minimize potential misclassification arising from intervention-induced changes rather than underlying frailty status while preserving the core functional domains of the frailty construct. Grip strength was operationalized using the maximum value across hands, and gait speed was derived from the average of repeated 4-meter walk measurements, with frailty classification based on the corresponding trial-derived indicators. Subjective components were based on Fried Function (i.e., self-report of limitations of walking half a mile) and Fried Effort (i.e., self-reported exhaustion) variables. For each phenotype measure, participants were categorized as non-frail (0 criterion), pre-frail (1–2 criteria) or frail (3 + criteria), based on the four assessed components. All frailty components were derived consistently at baseline and week 10 using the corresponding time-specific variables.

Change in frailty score was defined as:

$$\:\varDelta\:Frailty={Frailty}_{W10}-{Frailty}_{W0}$$

Negative values indicate improvement.

For the economic evaluation, a composite responder was defined as a participant demonstrating improvement in both HbA1c and frailty score between baseline and week 10. This composite endpoint was selected to reflect the co-occurrence of metabolic and functional risk in later life, rather than focusing on disease-specific change alone.

In addition, exploratory outcomes were examined to aid interpretation of quality-of-life differences and to assess whether the primary endpoint structure may differentially capture intervention effects. These included change in 12-Item Short Form Health Survey (SF-12) mental component score (MCS) and change in 30-second sit-to-stand performance, reflecting functionally proximal exercise-related benefit. The SF-12 is a brief, validated questionnaire that measures health-related quality of life across physical and mental health domains. It was developed as a shorter alternative to the SF-36, containing only 12 items while reproducing the Physical Component Summary (PCS) and MCS with high accuracy (R² of 0.91 and 0.92, respectively) [27]. The 30-second sit-to-stand test assesses lower extremity muscular endurance. Participants practiced one or two repetitions to ensure proper form and balance. Upon the signal “go,” participants were instructed to rise to a full standing position and then return to a fully seated position as many times as possible within 30 s. The test consisted of one trial [28]. These analyses were considered supplementary and interpretive rather than primary endpoints.

### Baseline and descriptive analyses

Baseline characteristics were summarized by intervention group using means and standard deviations for continuous variables and counts and percentages for categorical variables. Between-group differences were assessed using one-way analysis of variance (ANOVA) for continuous variables and chi-square tests for categorical variables, respectively (Table 1). The robustness of the composite responder comparison was evaluated using a Fisher–Freeman–Halton exact test as a sensitivity analysis because of the relatively small number of composite responders across the intervention groups.

**Table 1:** Baseline demographic and clinical characteristics of the analytic cohort of adults aged 65 years and older by intervention group

Characteristic	VegMedD:	Empagliflozin	Exercise:
Age, years: mean ± SD (N)	69.34 ± 3.91 (33)	71.64 ± 5.46 (33)	70.52 ± 4.29 (34)
Sex coded 1, n (%)	18 (54.5%)	22 (66.7%)	20 (58.8%)
BMI, kg/m²: mean ± SD (N)	32.24 ± 6.01 (33)	30.63 ± 6.23 (33)	32.08 ± 5.67 (34)
HbA1c, %: mean ± SD (N)	7.67 ± 1.37 (32)	7.57 ± 1.15 (32)	7.18 ± 0.83 (34)
Frailty score (operationalized phenotype): mean ± SD (N)	1.27 ± 0.88 (33)	1.21 ± 0.86 (33)	1.03 ± 1.09 (34)
Resting metabolic rate, kcal/day: mean ± SD (N)	1780.44 ± 306.06 (32)	1645.00 ± 337.87 (32)	1705.73 ± 218.52 (33)
Systolic blood pressure, mmHg: mean ± SD (N))	138.64 ± 13.76 (33)	137.40 ± 18.68 (33)	133.54 ± 12.49 (32)
Diastolic blood pressure, mmHg: mean ± SD (N)	74.32 ± 10.86 (33)	68.18 ± 12.12 (33)	71.55 ± 8.11 (32)

Continuous variables are presented as mean (SD), and categorical variables as number (%). Between-group comparisons were assessed using one-way analysis of variance (ANOVA) for continuous variables and chi-square tests for categorical variables. Frailty score reflects an operationalization based on exhaustion, functional status, grip strength, and gait speed, consistent with the available trial variables, excluding weight-loss and physical-activity components

*Abbreviations*: *BMI* Body mass index, *HbA1c* Hemoglobin A1c, *SD* Standard deviation

Descriptive comparisons of week-10 outcomes and change scores are reported in Table 2, with graphical representations of outcome changes provided in Supplementary Figures S1–S2.

**Table 2:** Week-10 metabolic and frailty outcomes, including change scores and composite responder status

Outcome	VegMedD:	Empagliflozin	Exercise:	*p*-value
HbA1c at week 10, %: mean ± SD (N)	7.06 ± 0.96 (29)	7.11 ± 0.89 (29)	6.94 ± 1.03 (26)	0.8053
Change in HbA1c (W10 − W0), %: mean ± SD (N)	-0.56 ± 0.93 (29)	-0.48 ± 0.92 (29)	-0.11 ± 0.52 (26)	0.1025
Frailty score at week 10: mean ± SD (N)	1.14 ± 0.93 (28)	0.78 ± 0.89 (27)	0.86 ± 0.77 (22)	0.2791
Change in frailty score (W10 − W0): mean ± SD (N)	-0.18 ± 0.82 (28)	-0.30 ± 0.91 (27)	-0.18 ± 0.91 (22)	0.8576
Composite responder, n/N (%): mean ± SD (N)	4/28 (14.3%)	8/27 (29.6%)	1/22 (4.5%)	0.0594

Frailty was operationalized using a four-component Fried-based score including exhaustion, functional status, grip strength, and gait speed, while excluding weight-loss and physical-activity components. Negative change values indicate improvement. Composite responder required improvement in both HbA1c and frailty score among complete cases. The composite responder comparison was evaluated using both the chi-square test and a Fisher–Freeman–Halton exact test as a sensitivity analysis because of the relatively small number of composite responders. The exact test yielded a similar non-significant result (*p* = 0.0633), and the overall statistical inference remained unchanged

*SD* Standard deviation

### Adjusted regression models

Adjusted intervention effects were estimated using analysis of covariance (ANCOVA) models. The general specification was:

$$\begin{aligned}{Y}_{i,10}=&{\beta\:}_{0}+{\beta\:}_{1}{Treatment}_{i}+{\beta\:}_{2}{Y}_{i,0}\\&+{\beta\:}_{3}{Age}_{i}+{\beta\:}_{4}{Sex}_{i}+{\epsilon}_{i}\end{aligned}$$

Separate models were estimated for HbA1c and frailty outcomes. Empagliflozin was used as the reference group. Results are reported in Supplementary Table S1.

Longitudinal changes were examined using linear mixed-effects models with participant-level random intercepts:

$$\begin{aligned}{Y}_{i,t}&={\beta\:}_{0}+{\beta\:}_{1}{Treatment}_{i}+{\beta\:}_{2}{Time}_{i}+{\beta\:}_{3}\\&({Treatment}_{i}\times\:{Time}_{i})+{u}_{i}+{\epsilon}_{it}\end{aligned}$$

Results are reported in Supplementary Table S2.

### Propensity score and weighted analyses

To assess robustness of treatment comparisons and address potential baseline imbalance, a multinomial propensity score model was estimated using age, sex, body mass index, baseline HbA1c, baseline frailty score, resting metabolic rate, systolic blood pressure, and diastolic blood pressure. The multinomial model is reported in Supplementary Table S3.

Inverse probability of treatment weighting with stabilized weights was applied to estimate weighted treatment effects. Results are reported in Supplementary Table S4.

Diagnostic plots assessing propensity score overlap and covariate balance are presented in Supplementary Figures S3–S4.

### Cost estimation

Intervention costs were estimated using official pharmaceutical price registries and published economic evaluations of dietary and exercise interventions. Drug costs were based on the Israeli Ministry of Health drug registry [29] and reflect official list prices for the 2024 price year, while diet and exercise costs were derived from published intervention studies (Appendix Table A1). Because pharmaceutical prices are dynamic and subject to reductions following patent expiry and generic entry, including in drug classes such as SGLT2 inhibitors, the base-case estimates reflect the observed price year but were interpreted alongside one-way, threshold, and scenario-based sensitivity analyses designed to explore plausible variations in pharmacological pricing conditions. This assumption is supported by recent evidence suggesting that several diabetes medications may be produced at substantially lower cost than current market prices [30]. All costs were expressed in Israeli Shekels (ILS) and reflect the 2024 price year. Detailed cost inputs and assumptions are presented in Appendix Table A1. The economic perspective adopted here is most closely aligned with direct intervention cost comparison over the trial horizon; accordingly, estimates should be interpreted as conservative with respect to broader downstream savings that may arise through prevention of disability, hospitalization, or long-term care use. To improve transparency, a detailed disaggregated description of intervention cost components is provided in the Supplementary Materials.

More specifically, the analysis was conducted from a restricted intervention-cost perspective, including direct intervention costs only. Accordingly, the present study represents a partial economic evaluation based on direct intervention costs. This approach was chosen to ensure comparability across intervention arms while minimizing reliance on non-observed assumptions and preserving internal validity of trial-based comparisons and was applied consistently across both cost-effectiveness and cost-utility analyses. Consequently, the analysis did not incorporate broader healthcare utilization, patient time, travel, informal care, or downstream healthcare costs and savings.

### Trial-based cost-effectiveness analysis

A trial-based cost-effectiveness analysis was conducted using the composite responder outcome. Incremental costs and effects were calculated relative to Empagliflozin using the composite responder definition based on the modified frailty operationalization.

$$\:ICER=\frac{\varDelta\:Cost}{\varDelta\:Effect}$$

Results are presented in Table 3, with net monetary benefit (NMB) estimates reported in Supplementary Table S5.

**Table 3:** Trial-based cost-effectiveness analysis using the composite responder outcome as the measure of effectiveness

Intervention	*N* randomized	Responder rate	Responders / complete cases	Mean cost (ILS)	Incremental cost vs. Empagliflozin	Incremental responder proportion vs. Empagliflozin	ICER (ILS per additional responder)
Empagliflozin	33	0.2963	8 / 27	608.9	Reference	Reference	Reference
Exercise	34	0.0455	1 / 22	197.2	-411.7	-0.2508	1641.27
VegMedD	33	0.1429	4 / 28	191.2	-417.7	-0.1534	2722.25

Costs are the arm-specific intervention costs used in the current model. The composite responder outcome was defined among complete cases only. ICERs are presented relative to Empagliflozin

The incremental responder proportion refers to the between-group difference in the proportion of participants achieving the composite responder outcome, defined as simultaneous improvement in both HbA1c and frailty score

*Abbreviations*: *ICER* Incremental cost-effectiveness ratio, *ILS* Israeli Shekels. Because responder counts were low in some arms, *ICER* Estimates should be interpreted with caution

Net monetary benefit (NMB) was calculated as:

$$\mathrm{NMB}=(\lambda\times\;\mathrm{incremental}\;\mathrm{effectiveness})-\mathrm{incremental}\;\mathrm{cost},$$

where *λ* represents the willingness-to-pay threshold. Positive NMB values indicate that an intervention provides greater economic value than the comparator at the specified willingness-to-pay threshold, whereas negative values indicate lower economic value. This framework facilitates decision-making by expressing health gains and costs on a common monetary scale and avoids some of the interpretative limitations associated with incremental cost-effectiveness ratios (ICERs) [31].

These estimates should be interpreted within the restricted intervention-cost perspective described above.

### Cost-utility analysis

Health-related quality of life was assessed using SF-12 data. Because SF-12 is not a preference-based instrument, utility values were estimated using a validated regression-based mapping approach from SF-12 physical and mental component scores (PCS and MCS) to preference-based utility values, as described by Hanmer [32]. To the best of our knowledge, no validated Israeli-specific mapping algorithm for deriving preference-based utility values from the SF-12 is currently available. Accordingly, the validated algorithm developed by Hanmer et al. was used as the most appropriate available approach for estimating utility values in the present study. QALYs were calculated using the area-under-the-curve method over the study period. This approach is widely used in economic evaluation when direct preference-based measures are unavailable. Although mapping introduces additional uncertainty compared with direct preference-based measurement, it enables consistent estimation of utility values within trial-based datasets.

$$\:QALY=\frac{{Utility}_{W0}+{Utility}_{W10}}{2}\times\:\frac{10}{52}$$

Utility and QALY estimates are reported in Supplementary Table S6, and incremental cost-utility results are reported in Supplementary Table S7. Because quality-adjusted outcomes may capture dimensions of health and functioning not fully reflected in disease-specific clinical endpoints, the cost-utility analysis was central to evaluating broader value in the context of healthy ageing.

This approach allowed the analysis to capture broader differences in perceived health status and functioning, while also acknowledging added uncertainty relative to direct preference-based measurement.

Because QALYs were estimated over only the 10-week trial period, they should be interpreted as reflecting short-term changes in health-related quality of life rather than long-term health benefit.

To aid interpretation of these quality-adjusted outcomes, supplementary analyses examined change in SF-12 MCS, given its conceptual proximity to perceived well-being and its contribution to utility-based measures.

### Uncertainty and sensitivity analyses

**Fig. 1:**
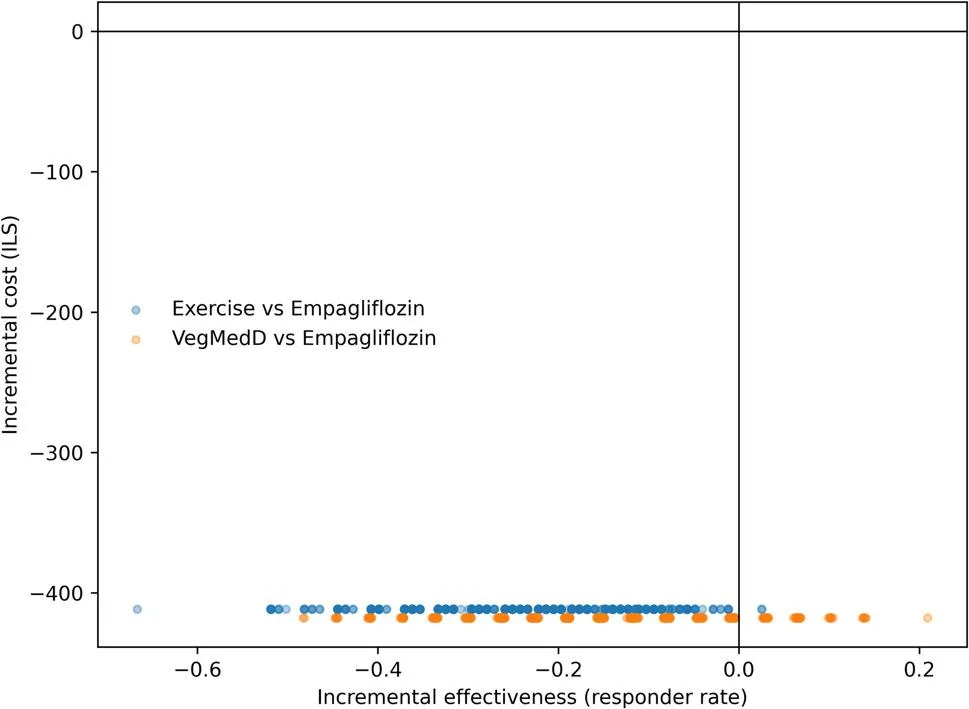
Cost-effectiveness plane illustrating the joint distribution of incremental costs and effects based on 1,000 non-parametric bootstrap replications. Note. Each point represents one bootstrap replication comparing an intervention strategy with Empagliflozin. The southeast quadrant indicates lower cost and greater effectiveness, the southwest quadrant indicates lower cost and lower effectiveness, the northeast quadrant indicates higher cost and greater effectiveness, and the northwest quadrant indicates higher cost and lower effectiveness. Effectiveness was defined using the composite responder outcome. Abbreviations. ILS = Israeli Shekels

**Fig. 2:**
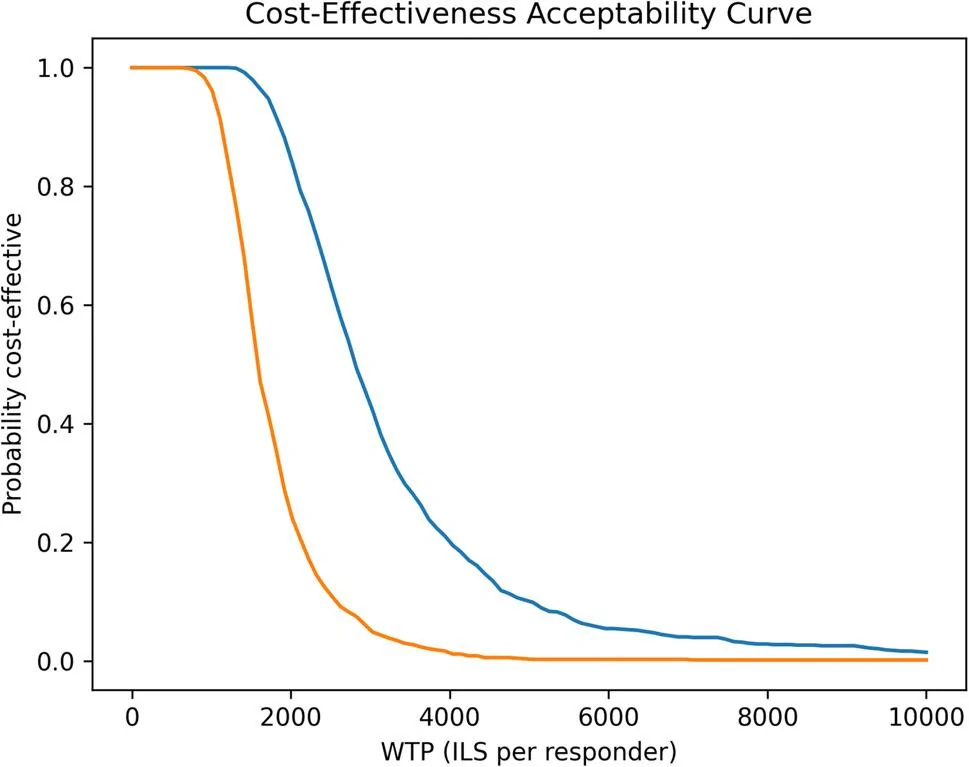
Cost-effectiveness acceptability curve showing the probability that each intervention is cost-effective relative to Empagliflozin across willingness-to-pay thresholds ranging from 0 to 10,000 ILS per composite responder. Note. Probabilities were estimated from 1,000 bootstrap replications using the net monetary benefit framework. The curves summarize decision uncertainty across alternative willingness-to-pay thresholds based on the composite responder outcome. Abbreviations. CEAC = Cost-effectiveness acceptability curve; ILS = Israeli Shekels

Uncertainty was explored using non-parametric bootstrap resampling (1,000 iterations). The distribution of incremental costs and effects is presented in the cost-effectiveness plane (Fig. 1), and cost-effectiveness probabilities across willingness-to-pay thresholds are summarized in the CEAC (Fig. 2). Numerical bootstrap results are reported in Appendix Table A3.

Probabilistic sensitivity analysis assigned Gamma distributions to costs and Beta distributions to probabilities and utilities. Parameter distributions are provided in Appendix Table A2.

One-way deterministic sensitivity analysis (OWSA) was conducted by varying key model parameters individually, including intervention costs, to evaluate the robustness of the economic findings to parameter uncertainty. Results are presented in Supplementary Figure S5. To further interpret quality-of-life differences, exploratory analyses are presented in Supplementary Figures S6–S7. Supplementary Figure S6 presents changes in SF-12 MCS, and Supplementary Figure S7 presents results for 30-second sit-to-stand performance.

In addition, cost-utility uncertainty was examined using patient-level QALY estimates derived from SF-12 data, with non-parametric bootstrap resampling applied within each intervention group. Results are reported in Supplementary Table S11 and Supplementary Figure S8.

Additional one-way deterministic sensitivity analyses explored uncertainty in pharmacological pricing by varying the 10-week cost of empagliflozin, performing threshold analyses, and evaluating a class-equivalent SGLT2 pricing scenario. Results are reported in Supplementary Tables S12–S14 and Supplementary Figures S9–S10.

### Reproducibility

All statistical analyses were implemented through scripted procedures to ensure full computational reproducibility. The economic evaluation followed standard methodological principles for trial-based cost-effectiveness analysis.

All analyses were conducted using Python version 3.13.5, using pandas, numpy, scipy, statsmodels, scikit-learn, matplotlib, and openpyxl.

The replication package includes all scripts and outputs required to reproduce:

- Tables 1, 2 and 3.
- Supplementary Tables S1–S14.
- Appendix Tables A1–A3.
- Figures 1, 2 and Supplementary Figures S1–S8.

## Results

### Baseline characteristics

Baseline characteristics of the analytic cohort are presented in Table 1. The study included 100 participants distributed across three intervention groups: VegMedD, Empagliflozin, and Exercise. The groups were broadly comparable at baseline with respect to age, sex distribution, body mass index, glycemic status (HbA1c), frailty score, resting metabolic rate, and blood pressure. No statistically significant between-group differences were observed for any of these baseline variables (all *p* > 0.05), indicating no clear evidence of systematic baseline imbalance.

### Clinical outcomes

Week-10 outcomes and changes from baseline are summarized in Table 2. All intervention groups demonstrated reductions in HbA1c, with larger mean decreases observed in the VegMedD and Empagliflozin groups than in the Exercise group. Mean change in HbA1c was − 0.56% in VegMedD, − 0.48% in Empagliflozin, and − 0.11% in Exercise, although between-group differences did not reach statistical significance (*p* = 0.1025).

Mean frailty score improved modestly across all intervention groups, with no statistically significant between-group differences (*p* = 0.8576). The composite responder outcome showed the highest proportion of responders in the Empagliflozin group (29.6%), followed by VegMedD (14.3%) and Exercise (4.5%), with no statistically significant differences between groups (*p* = 0.0594). Taken together, these findings suggest broad short-term clinical similarity across intervention types, although pharmacological treatment demonstrated a numerically higher responder rate.

Adjusted analyses are presented in Supplementary Table S1 and Supplementary Table S2. Specifically, Supplementary Table S1 reports analysis of covariance models for week-10 HbA1c and frailty outcomes, and Supplementary Table S2 reports mixed-effects models for repeated measures from baseline to week 10. In both modeling approaches, baseline values were strong predictors of follow-up outcomes, while adjusted between-group differences remained non-significant. This pattern strengthens the interpretation that the main distinction between intervention strategies in this study lies less in short-term clinical effect size than in their economic profile and broader implications for value.

Clinical outcome distributions by intervention group are additionally illustrated in Supplementary Figure S1 (change in HbA1c) and Supplementary Figure S2 (change in Fried frailty score).

### Propensity score and weighted analyses

Because residual baseline imbalance may still affect treatment comparisons even when crude baseline differences are not statistically significant, robustness analyses based on propensity score methods were conducted before the economic results are presented. The multinomial propensity score model is reported in Supplementary Table S3. Most baseline covariates were not significantly associated with treatment assignment, suggesting limited systematic differences between groups after accounting for observed characteristics.

Inverse probability of treatment weighting results are reported in Supplementary Table S4. After weighting, estimated treatment effects on HbA1c and frailty outcomes at week 10 remained non-significant, consistent with the primary and adjusted analyses.

Diagnostic plots are presented in Supplementary Figure S3 (propensity score overlap) and Supplementary Figure S4 (covariate balance), indicating adequate overlap and improved covariate balance following weighting.

### Economic evaluation

#### Trial-based cost-effectiveness analysis

Trial-based cost-effectiveness results are presented in Table 3. Mean intervention costs were substantially lower for VegMedD (191.2 ILS) and Exercise (197.2 ILS) than for Empagliflozin (608.9 ILS). Composite responder rates were highest in the Empagliflozin group (29.6%), lowest in the Exercise group (4.5%), and intermediate in VegMedD (14.3%).

Relative to Empagliflozin, both VegMedD and Exercise were associated with lower costs but also lower effectiveness. The corresponding incremental cost-effectiveness ratios (ICERs) were 2,722.25 ILS and 1,641.27 ILS per additional responder lost for VegMedD and Exercise, respectively. These estimates indicate that differences between interventions are modest in magnitude, although the overall pattern of higher effectiveness but higher cost for pharmacological treatment remains unchanged.

These ICERs should be interpreted with caution. In particular, the very low responder count in the Exercise arm (1/24 complete cases) indicates that the incremental effect estimate is driven by a single observed responder, making the resulting ICER highly sensitive to small-sample variation and limiting its reliability for inference. Accordingly, greater interpretive weight should be placed on the probabilistic sensitivity analyses, net monetary benefit estimates, and cost-effectiveness acceptability curves than on the deterministic ICER point estimates, particularly given the limited number of responders observed in the Exercise group.

Net monetary benefit (NMB) estimates across willingness-to-pay thresholds are reported in Supplementary Table S5, indicating that Empagliflozin yielded the highest NMB at λ = 5,000 ILS, followed by VegMedD and Exercise. Thus, when effectiveness is defined narrowly through the composite responder metric, pharmacological treatment is economically preferred despite higher intervention costs, reflecting its higher observed responder rate in the present sample.

Additional scenario analyses indicated that responder-based economic conclusions were sensitive to pharmacological pricing assumptions. As the assumed cost of empagliflozin decreased, its net monetary benefit increased relative to lifestyle-based interventions. In a class-equivalent SGLT2 scenario based on a lower-cost pharmacological alternative, the pharmacological arm remained favored under the responder-based framework. Threshold analysis further showed that, at a willingness-to-pay threshold of 5,000 ILS per responder, empagliflozin would retain higher net monetary benefit than VegMedD unless its 10-week cost exceeded 958.40 ILS, and higher net monetary benefit than Exercise unless its cost exceeded 1,451.41 ILS (Supplementary Tables S12–S14).

#### Cost-utility analysis

Over the 10-week study period, mean QALYs were highest in the VegMedD group (0.1536 ± 0.0154), followed by Exercise (0.1502 ± 0.0200), and lowest in the Empagliflozin group (0.1481 ± 0.0208), as shown in Supplementary Table S6. Cost-utility results are reported in Supplementary Table S7. Relative to Empagliflozin, both VegMedD and Exercise were associated with lower costs and higher QALYs, indicating that these interventions were economically dominant within the short-term analytic horizon and under current pharmacological pricing conditions. These results were derived from patient-level utility estimates and are fully consistent with the probabilistic sensitivity analysis presented in Supplementary Table S11 and Supplementary Figure S8, which are based on the same underlying individual-level QALY data and cost distributions. Because these QALY estimates were derived exclusively from the randomized 10-week follow-up period, they represent short-term changes in health-related quality of life and should not be interpreted as estimates of longer-term health benefit or comparative intervention value. This contrast between the responder-based and utility-based findings suggests that the choice of evaluative framework materially affects how intervention value is interpreted. It also suggests that broader outcome measures may capture dimensions of benefit that are less visible when effectiveness is defined only through short-term composite clinical response. In contrast, cost–utility conclusions were substantially more robust to lower pharmacological price assumptions. Across all one-way sensitivity scenarios, including the class-equivalent SGLT2 scenario, VegMedD retained the highest net monetary benefit at conventional willingness-to-pay thresholds per QALY. Threshold analysis further indicated that, at commonly used QALY thresholds, no plausible positive empagliflozin price would reverse the ranking relative to VegMedD, reflecting the combined effect of higher costs and lower QALYs observed for the pharmacological arm in the present sample (Supplementary Tables S12–S14).

The detailed cost inputs and assumptions underlying the economic evaluation are presented in Appendix Table A1, while parameter distributions used in probabilistic sensitivity analysis are presented in Appendix Table A2.

To further interpret the quality-of-life findings, additional exploratory analyses are presented in Supplementary Figures S6–S7 and Supplementary Tables S8–S9. Supplementary Table S8 and Supplementary Figure S6 report change in SF-12 mental component score, while Supplementary Table S9 and Supplementary Figure S7 present results for 30-second sit-to-stand performance.

Notably, the mental component summary results (Supplementary Table S8 and Figure S6) demonstrate a statistically significant improvement in perceived well-being in the VegMedD and Exercise groups compared with a decline in the Empagliflozin group (*p* = 0.0466), providing a clinically plausible explanation for the observed short-term differences in QALY estimates.

Similarly, the 30-second sit-to-stand results (Supplementary Table S9 and Figure S7) indicate superior functional improvement in the Exercise group, supporting the interpretation that utility gains reflect meaningful changes in functional capacity rather than measurement noise.

These analyses suggest that quality-of-life differences across interventions may reflect clinically meaningful differences in perceived well-being and functional capacity that are not fully captured by the composite responder definition. Together, these findings reinforce the interpretation that QALY-based evaluation captures broader dimensions of value, including subjective well-being and functional performance, which are central to healthy ageing but underrepresented in conventional clinical endpoints.

A disaggregated summary of intervention cost assumptions is provided in Supplementary Table S10.

#### Economic uncertainty analysis

Uncertainty surrounding the economic evaluation was explored using non-parametric bootstrap resampling (1,000 iterations). The resulting joint distribution of incremental costs and effects is illustrated in the cost-effectiveness plane (Fig. 1).

At a willingness-to-pay threshold of 5,000 ILS per responder, VegMedD had an approximately 32% probability of being cost-effective, whereas Exercise had a substantially lower probability (approximately 4%).

Full bootstrap-based cost-effectiveness results are presented in Appendix Table A3. Bootstrap-based ICER estimates were consistent with the base-case analysis, with minor variation reflecting resampling uncertainty.

These findings are summarized in the cost-effectiveness acceptability curve (Fig. 2), which presents the probability that each intervention is economically preferred across willingness-to-pay thresholds ranging from 0 to 10,000 ILS. Results of deterministic sensitivity analysis are presented in Supplementary Figure S5. Cost-utility uncertainty results are additionally presented in Supplementary Table S11 and Supplementary Figure S8. These patient-level probabilistic analyses indicate that VegMedD maintains a very high and stable probability of cost-effectiveness across a wide range of willingness-to-pay thresholds per QALY, whereas the exercise-based intervention also shows a high probability of cost-effectiveness, albeit with greater variability across thresholds.

To further interpret the divergence between responder-based and utility-based findings, exploratory analyses of quality-of-life and functional outcomes are presented in Supplementary Tables S8–S9 and Supplementary Figures S6–S7. These analyses suggest that differences in QALYs may reflect variation in perceived well-being and functional capacity that are not fully captured by the composite responder outcome.

Taken together, these findings indicate that uncertainty in economic conclusions depends on the outcome definition: responder-based analyses suggest modest and uncertain effectiveness differences, whereas utility-based analyses consistently favor non-pharmacological interventions when broader quality-of-life outcomes are considered.

Additional cost scenario analyses are presented in Supplementary Tables S12–S14 and Supplementary Figures S9–S10. These analyses extend the uncertainty framework by incorporating variation in pharmacological pricing, complementing the probabilistic sensitivity analyses based on clinical and utility variation.

## Discussion

### Principal findings

Across three intervention strategies in older adults, we observed broadly similar short-term clinical outcomes but substantial divergence in economic value. All interventions produced modest improvements in glycemic control and frailty, with no statistically significant between-group differences. However, pharmacological treatment achieved the highest responder rate, whereas dietary and exercise-based interventions were consistently less costly. When value was assessed using cost–utility analysis, both non-pharmacological strategies were economically dominant within the short-term analytic framework applied in this study and the observed trial horizon, yielding lower costs alongside higher QALYs compared to pharmacological treatment. Notably, the dietary intervention demonstrated the highest probability of cost-effectiveness across conventional willingness-to-pay thresholds. Supplementary analyses of mental well-being and functionally proximal outcomes further suggest that the utility advantage of the lifestyle interventions is clinically interpretable rather than arbitrary and is supported by both subjective MCS and objective (functional performance) supplementary outcomes.

These findings suggest that interventions appearing clinically comparable in the short term may nonetheless differ meaningfully in their broader value for ageing populations. The results therefore highlight a central tension in contemporary health systems: reliance on narrow clinical endpoints may obscure important differences in economic efficiency, quality of life, and long-term system sustainability. By integrating clinical, economic, and population health perspectives within a single analytic framework, this study shows that lifestyle-based interventions may offer a more favorable short-term value profile within utility-based evaluation frameworks and short-term trial horizons relevant to healthy ageing. These findings should not be interpreted as indicating superiority of lifestyle interventions over pharmacological treatment, but rather as reflecting differences in short-term value under specific analytic assumptions. Although the observed between-group differences in QALYs were modest, this is expected given the short 10-week follow-up period and the limited opportunity for substantial changes in health-related quality of life over such a timeframe. In trial-based economic evaluations, even small incremental QALY gains may have meaningful implications when accompanied by substantial differences in intervention costs, because cost-effectiveness depends on the joint consideration of costs and health outcomes rather than on the magnitude of QALY differences alone. Accordingly, the interpretation of the present findings should focus on the combined economic and clinical evidence rather than on incremental QALYs in isolation. This interpretation is also supported by the probabilistic sensitivity analyses, which demonstrated favorable economic value despite the relatively small absolute differences in QALYs. The present trial-based economic evaluation was intentionally restricted to the observed 10-week randomized trial period. Consequently, the findings should be interpreted as exploratory estimates of short-term economic value rather than as comprehensive estimates of long-term cost-effectiveness. This distinction is particularly important for empagliflozin and other SGLT2 inhibitors, whose established cardiovascular and renal benefits generally emerge over substantially longer follow-up periods than those evaluated in the present study. Accordingly, the present findings should not be interpreted as challenging the established long-term clinical value of SGLT2 inhibitors, but rather as comparing the short-term economic performance of alternative interventions within the observed 10-week randomized trial period.

This study further contributes to social science and population health literature by demonstrating how different models of care shape value beyond traditional biomedical evaluation. More specifically, the study shows that interventions appearing broadly similar on short-term clinical endpoints may diverge meaningfully once value is assessed in terms of costs, quality of life, and health system relevance. This is an important distinction for ageing policy, where the question is often not which intervention produces the largest short-term biomarker change, but which intervention generates the most meaningful and sustainable value for older populations. In this sense, the study’s central message may be summarized as one of clinical similarity but economic divergence: interventions that look broadly comparable in short-term biomedical terms may still differ meaningfully in their wider societal value. This pattern is consistent with the broader literature demonstrating that economic conclusions depend critically on analytic horizon and outcome definition. These findings should be interpreted in the context of existing economic evidence, which has not been uniform in its conclusions regarding the relative value of pharmacological and lifestyle-based interventions. Notably, findings from the Diabetes Prevention Program (DPP) demonstrated that pharmacological treatment with metformin may be cost-effective or even cost-saving over extended time horizons, particularly in the context of diabetes prevention [22]. These results differ from the present findings, in which non-pharmacological interventions were associated with more favorable cost–utility profiles within the short-term analytic horizon and evaluation framework applied in this study. However, this apparent discrepancy is likely explained by important differences in study design, population, intervention structure, and analytic time horizon. Importantly, these differences do not imply inferiority of pharmacological therapy but reflect differences in evaluative scope and time horizon.

First, the DPP was conducted in a pre-diabetic population and evaluated long-term outcomes over several years, allowing the capture of downstream cost savings related to diabetes prevention and complication avoidance. In contrast, the present study focuses on older adults with established type 2 diabetes and evaluates outcomes over a short-term, trial-based horizon, where long-term benefits of pharmacological therapy may not yet be fully realized. Second, the lifestyle intervention in the DPP was highly intensive and resource-demanding, whereas the interventions evaluated here were pragmatic and lower-cost, which substantially affects comparative cost structures. Third, the current analysis incorporates both responder-based and QALY-based frameworks, demonstrating that conclusions regarding intervention value depend not only on cost assumptions but also on the choice of evaluative metric.

Importantly, scenario and threshold analyses conducted in the present study indicate that responder-based cost-effectiveness conclusions are sensitive to pharmacological pricing assumptions, particularly when considering future price reductions within the SGLT2 inhibitor class following patent expiry and increased generic availability. Under lower-cost scenarios, pharmacological strategies may become more economically competitive. However, cost–utility results based on QALY outcomes remained relatively stable within the explored parameter ranges, consistently favoring lifestyle-based interventions.

Taken together, these findings highlight that economic conclusions in chronic disease management are highly context-dependent and sensitive to analytic perspective, time horizon, and outcome definition.

An important interpretive consideration is the dynamic nature of pharmaceutical pricing. The present base-case analysis reflects the observed price year and is therefore appropriate for a trial-based economic evaluation. However, future price reductions following patent expiry and generic entry may narrow the cost differences observed here within the parameter ranges explored in the sensitivity analyses. As generic SGLT2 inhibitors become increasingly available following patent expiry, the current cost advantage of lifestyle-based interventions over empagliflozin may therefore narrow over time, even if differences in health outcomes remain unchanged. The additional scenario analyses suggest that such reductions would strengthen the pharmacological arm under a responder-based framework but are unlikely to fully overturn the broader cost–utility findings within the observed parameter ranges, which incorporate differences in quality-adjusted outcomes, perceived well-being, and functional performance. These conclusions are supported by structured sensitivity analyses (Supplementary Tables S12–S14), which demonstrate that while pharmacological cost reductions improve responder-based economic attractiveness, they do not appear to materially alter the ranking under utility-based evaluation frameworks. In addition, it is important to emphasize that the present analysis does not imply replacement of pharmacological therapy with lifestyle-based interventions. Rather, the findings should be interpreted within a contemporary clinical framework in which pharmacological treatment remains a central component of diabetes management, and lifestyle interventions are best understood as complementary strategies that may enhance overall value, particularly in domains of quality of life, functioning, and patient-centered outcomes. Furthermore, the established cardiovascular and renal benefits of SGLT2 inhibitors should be explicitly considered when interpreting the findings. These long-term cardiovascular and renal benefits, which are well established in the literature, are not fully captured within the short-term analytic horizon of the present study. As such, the current results reflect a partial view of intervention value, focused primarily on short-term outcomes. As a result, the overall clinical value of pharmacological treatment may be underestimated in short-term economic evaluations.

To clarify the policy relevance of these findings, it is important to recognize that different evaluative frameworks may lead to different intervention rankings. Under a responder-based cost-effectiveness framework, pharmacological treatment may appear preferable due to higher short-term clinical response rates. In contrast, under a cost–utility framework incorporating quality-adjusted life years, lifestyle-based interventions may offer greater overall value within the short-term analytic horizon. These findings do not represent a contradiction but rather illustrate how the choice of outcome measure and evaluative framework shapes conclusions regarding value.

### Clinical outcomes in the context of ageing, multimorbidity, and life-course processes

The observed improvements in glycemic control across all intervention groups, without statistically significant differences between them, suggest that multiple therapeutic pathways may achieve comparable short-term metabolic outcomes in older adults. From a life-course perspective, this reflects the cumulative nature of chronic disease processes, where late-life interventions often yield incremental rather than transformative effects, particularly in populations characterized by multimorbidity and functional heterogeneity [10]. Similarly, the modest improvements in frailty highlight the multidimensional nature of functional decline in later life. Frailty emerges not only from biological ageing but also from accumulated social and environmental exposures, including socioeconomic disadvantage, nutrition, and access to care [4, 5]. Recent international evidence also indicates that frailty is highly prevalent across settings and varies across populations, reinforcing the importance of viewing frailty through a broader population health lens [9].

The absence of significant between-group differences across multiple analytic approaches reinforces the interpretation that short-term intervention effects may be constrained by these broader determinants and by the limited time horizon over which change was observed.

This interpretation should also be considered in light of how frailty was operationalized in the present study. While key components such as grip strength and gait speed were directly measured, other elements may partly reflect intervention-induced effects rather than underlying vulnerability processes, particularly in domains directly targeted by the interventions, such as metabolic adaptation and short-term functional responses. In addition, the relatively short follow-up period may further limit the extent to which changes in frailty capture longer-term physiological vulnerability rather than transient intervention effects. As a result, short-term changes in the composite frailty score should be interpreted cautiously, especially in intervention settings where metabolic and behavioral changes are expected.

Future studies incorporating the complete Fried phenotype or alternative multidimensional frailty instruments should examine whether inclusion of all frailty domains influences comparative estimates of intervention effectiveness.

The 10-week follow-up window is likely more suitable for detecting glycemic change than for capturing the full functional effects of exercise on frailty-related performance domains, particularly in older adults with vulnerability to sarcopenia and reduced physical reserve.

These findings should not be read as evidence that intervention modality is irrelevant. Rather, they suggest that in older populations with intertwined metabolic and functional vulnerability, short-term clinical equivalence may be common, particularly when interventions operate on pathways that differ in timing, intensity, and mechanism. This makes the broader evaluative lens of economic and population health analysis especially important. At the same time, the supplementary analyses indicate that endpoint choice may influence comparative interpretation. Because HbA1c is inherently more proximal to metabolic intervention effects, and because the exercise intervention evaluated here was pragmatic and home-based rather than intensive supervised resistance training, the primary composite endpoint may have favored dietary and pharmacological mechanisms more directly than exercise-related functional pathways. This asymmetry in endpoint proximity may contribute to the observed divergence between clinical and utility-based findings and underscores the importance of aligning outcome selection with intervention mechanisms when interpreting comparative effectiveness.

### Reframing value through a capability and well-being lens

While clinical outcomes were similar, the economic findings reveal substantial differences in value across interventions. Non-pharmacological strategies, particularly dietary interventions, were associated with lower costs and, in cost-utility terms, higher QALYs, indicating economic dominance within the cost–utility framework applied in this study and the observed trial horizon. From a capability approach perspective (Sen, 1999), these findings are particularly relevant. Health should be understood not only in terms of clinical indicators but also in terms of individuals’ ability to function, maintain autonomy, and participate in society. This perspective is especially important in ageing populations, where preserving independence and everyday functioning may be at least as important as achieving marginal improvements in disease-specific biomarkers. Relatedly, distributional and equity-sensitive approaches to economic evaluation have emphasized that intervention value should be assessed not only in aggregate terms but also in relation to broader questions of well-being and fairness [23].

Lifestyle-based interventions may therefore generate additional benefits that extend beyond biomedical disease control. Dietary and exercise interventions may influence physical functioning, self-management, and social participation, all of which are central to healthy ageing frameworks even when short-term clinical endpoints show only modest differences. In this sense, the contrast between the responder-based cost-effectiveness results and the QALY-based cost-utility results is theoretically informative rather than merely technical: it suggests that the choice of outcome measure may influence how intervention value is interpreted. If evaluation is restricted to narrow clinical response metrics, the benefits of lifestyle-based interventions may appear limited; if broader quality-of-life and functioning-oriented measures are incorporated, a different hierarchy of value emerges.

Importantly, this divergence is consistent with the measurement dynamics discussed above. When primary endpoints are more proximal to specific intervention mechanisms (e.g., HbA1c for metabolic interventions), they may systematically under-represent benefits that accrue through alternative pathways, such as improvements in perceived well-being, functional engagement, or self-efficacy. In this context, QALY-based outcomes may better capture dimensions of value that are more aligned with the lived experience of ageing than biomarker-based endpoints alone.

At the same time, the responder-based findings should be interpreted cautiously because low event counts, particularly in the exercise group, make these estimates more vulnerable to instability than the broader utility-based comparisons. Consequently, the deterministic ICER estimates should be interpreted primarily as descriptive summaries of the observed trial data, whereas the probabilistic sensitivity analyses provide a more informative assessment of decision uncertainty.

This interpretation is further supported by the sensitivity analyses, which indicate that conclusions are driven more by cost structure than by small differences in clinical effectiveness. Specifically, one-way and threshold analyses (Supplementary Tables S12–S13) show that responder-based conclusions are sensitive to pharmacological pricing, whereas cost–utility results remain comparatively stable within the explored parameter ranges.

It is also reinforced by the supplementary analyses (Supplementary Tables S8–S10; Figures S6–S7), which demonstrate that lifestyle-based interventions were associated with improvements or favorable trends in mental well-being and functional performance, even in the absence of statistically significant differences in primary clinical endpoints. These findings provide a plausible mechanism for the observed QALY advantage and suggest that utility-based measures may be more sensitive to short-term experiential and functional changes in older adults.

Finally, patient-level probabilistic sensitivity analysis of QALY outcomes (Supplementary Table S11; Figure S8) further supports this interpretation, showing that the dietary intervention maintains a consistently high probability of cost-effectiveness across conventional willingness-to-pay thresholds, while exercise-based strategies exhibit greater variability.

This pattern is consistent with the class-equivalent SGLT2 pricing scenario (Supplementary Table S14), in which pharmacological cost reductions do not fully eliminate the relative advantage observed within the utility-based evaluation framework.

### Social determinants, inequality, and differential access

A central implication of this study concerns the interaction between intervention effectiveness and social determinants of health. While non-pharmacological interventions appear economically advantageous, their real-world impact is likely contingent on access to enabling resources. Structural factors including income, education, neighborhood conditions, and food environments play a critical role in shaping individuals’ ability to adopt and sustain lifestyle changes. This concern is closely aligned with the wider evidence base on health equity and ageing, which emphasizes that socioeconomic disadvantage becomes biologically and functionally embodied across the life course [8, 33].

This also raises an important equity concern. Interventions designed to improve diet and physical activity are not always equally effective across socioeconomic groups. A systematic review by McGill et al. found that some “downstream” healthy-eating interventions may risk widening inequalities, whereas more structural or “upstream” approaches may be more likely to reduce them [24].

Accordingly, the policy relevance of lifestyle-based interventions depends not only on their average cost-effectiveness but also on whether health systems create the material and social conditions required for equitable uptake. Effective implementation requires not only individual motivation but also supportive environments, affordable healthy food, accessible community infrastructure, and delivery models that do not assume equal baseline capacity across all groups. In other words, the social distribution of capability conditions may determine whether the economic promise of lifestyle-based care is realized in practice or confined to relatively advantaged populations.

### Health systems transformation and sustainability

The findings may cautiously support consideration of a partial reorientation of health systems toward preventive, community-based models of care. However, these results do not in themselves constitute sufficient evidence for system-level transformation, given the short-term nature of the evidence and the absence of long-term outcome data.

The economic dominance of non-pharmacological interventions within the cost–utility framework applied in this study suggests that short-term gains in patient-centered outcomes may be achieved through relatively low-cost strategies that operate outside traditional clinical settings. This interpretation is consistent with the World Health organization Decade of Healthy Ageing framework [2], which emphasizes functional ability, supportive environments, and person-centered systems rather than disease treatment alone. In ageing societies, where healthcare demand is rising rapidly, strategies that preserve functioning at relatively low cost may be particularly important for long-term system sustainability.

At the same time, the findings should be interpreted within the context of current clinical practice, in which pharmacological treatment remains a central component of care. While pharmacological interventions are essential in many contexts, they may not fully address the broader determinants of health that shape later-life trajectories. In this context, integrating lifestyle-based interventions into standard care pathways may represent a complementary and potentially more holistic model of care. More broadly, the results suggest that routine resource allocation may undervalue interventions, such as nutrition and exercise, that improve everyday functioning and quality of life, particularly when these outcomes are less visible in conventional clinical reporting. However, because the present costing approach included only direct intervention costs, this interpretation is likely conservative within the analytic framework applied.

This conservatism is further supported by structured scenario and threshold analyses (as demonstrated in the scenario and threshold analyses), which indicate that even under substantially reduced pharmacological pricing, the relative economic advantage of lifestyle-based interventions appears to persist within the explored parameter ranges when broader quality-of-life and functional outcomes are considered. However, these outcomes are based on short-term observations, and their persistence over longer time horizons remains uncertain and cannot be established based on the present short-term evidence.

If broader downstream consequences such as hospitalization, disability, caregiving burden, or long-term care utilization were incorporated, the relative economic attractiveness of lifestyle-based interventions could differ across domains, particularly those related to functioning and patient-reported outcomes.

However, long-term comparative effects remain uncertain, and existing evidence suggests that pharmacological interventions may confer substantial benefits in preventing downstream clinical events.

At the same time, the supplementary cost breakdown highlights that the exercise-arm estimate reflects the pragmatic home-based format evaluated in this study rather than the cost of intensive supervised exercise programs, a distinction that is important for external interpretation. The economic evaluation was intentionally restricted to intervention-delivery costs expected under routine implementation. Consequently, research-specific activities associated with conducting the randomized trial (e.g., recruitment, randomization, protocol administration, data management, and study coordination) were not included because they do not represent routine clinical practice. Therefore, the reported cost estimates should be interpreted as intervention-delivery costs rather than as the full costs of conducting the randomized trial.

### Uncertainty, context, and implementation

The probabilistic sensitivity analyses highlight the role of uncertainty in economic evaluation within the context of this study. While dietary interventions demonstrate a relatively high probability of cost-effectiveness, exercise-based interventions exhibit greater variability across simulations. This pattern is consistent with the interpretation that intervention value is context-dependent and influenced by factors such as adherence, program design, and population characteristics. From a systems perspective, it may therefore be useful to consider interventions not as fixed packages with uniform effects, but as components introduced into dynamic social and organizational contexts, where outcomes depend partly on local conditions, relationships, and adaptive processes [34]. This interpretation is particularly relevant for lifestyle-based interventions, whose real-world effectiveness is likely to depend on implementation quality and social embedding.

The supplementary cost-utility uncertainty results further support the interpretation that broader evaluative conclusions may differ from responder-based rankings, particularly when utility-based measures capture dimensions of well-being that are not fully reflected in narrow clinical composites.

Additional pricing scenario analyses (Supplementary Tables S12–S14) extend this uncertainty framework by illustrating that economic conclusions may also be sensitive to evolving cost structures within pharmacological classes, including well-established, predictable, and near-universal patterns of price reduction following patent expiry and market competition.

### Methodological contribution

This study advances methodological practice by integrating clinical, economic, and robustness analyses within a unified framework, including propensity score weighting and probabilistic sensitivity analysis. This approach is consistent with broader calls for decision frameworks that consider not only efficiency but also distributional consequences and population health relevance [23]. Methodologically, the study also illustrates the importance of combining responder-based and utility-based analyses in ageing research, where interventions may generate only modest short-term clinical effects yet still alter quality-adjusted outcomes in policy-relevant ways. It also shows the analytical value of examining how conclusions change when effectiveness is defined narrowly versus more broadly, particularly in older populations where functioning and quality of life are central policy concerns.

The supplementary analyses strengthen this contribution by showing that the choice of endpoint structure is not merely technical but may materially shape comparative rankings across intervention types.

In this context, it is important to recognize that different evaluative frameworks may capture distinct dimensions of value. While utility-based measures reflect patient-reported outcomes and quality of life, they may be more sensitive to short-term behavioral changes, whereas clinical endpoints and long-term outcomes may better capture the sustained effects of pharmacological interventions.

### Policy Implications

The policy implications of these findings are potentially important. First, they suggest that health systems serving ageing populations should not rely exclusively on disease-specific clinical endpoints when prioritizing interventions. When clinical differences are modest, broader evaluative frameworks, particularly those incorporating quality of life, functioning, and cost, may offer a more policy-relevant basis for decision-making. This is especially relevant in older adults with diabetes, where treatment goals commonly extend beyond glycemic control alone.

However, the observed improvements in quality of life should be interpreted in the context of the relatively short follow-up period. These effects may reflect short-term behavioral adaptation and increased engagement associated with lifestyle changes, and their persistence over longer time horizons remains uncertain.

Importantly, these findings should not be interpreted as suggesting that lifestyle interventions can substitute pharmacological treatment in routine clinical practice. Rather, they should be interpreted as reflecting differences in short-term value under specific analytic assumptions.

Second, the results support consideration of increased attention to preventive and community-based models of care. Dietary and exercise-oriented strategies may represent relatively low-cost ways of improving outcomes that matter to older adults, particularly when policy goals extend beyond disease control to healthy ageing, independence, and social participation.

Moreover, because the present analysis adopts a restricted intervention-cost perspective, the economic advantage of lifestyle-based strategies is likely conservative and should be interpreted alongside the likelihood of future pharmaceutical price reductions.

From a dynamic health economic perspective, pharmacological costs are expected to decline substantially over time following patent expiration and market competition. Therefore, the relative cost advantage observed for lifestyle interventions under current pricing conditions should be understood as time-dependent rather than fixed.

Scenario analyses indicate that lower SGLT2 prices may improve the relative responder-based cost-effectiveness of pharmacological treatment but are unlikely to fully eliminate the broader quality-of-life and functional advantages observed for lifestyle-based interventions within the evaluation framework applied. This distinction is explicitly demonstrated in the scenario analyses, which show that while pharmacological price reductions may shift conclusions under responder-based frameworks, they do not materially alter the ranking of interventions when evaluated using QALY-based outcomes within the observed parameter ranges.

Third, equity considerations should be treated as central rather than peripheral. Lifestyle-based interventions may appear economically attractive, but their real-world success depends on whether populations have the resources required to engage with them. Policies that subsidize healthy food access, strengthen local physical activity infrastructure, and embed preventive services within accessible primary care and community systems are likely to improve both effectiveness and fairness.

Finally, the findings imply that reimbursement and commissioning structures may warrant reconsideration. If current systems disproportionately reward pharmacological management while underfunding preventive, lifestyle-based care, economically valuable strategies may remain underused despite their broader societal benefits.

From a policy perspective, future decision-making should account for both the potential decline in pharmacological costs over time and their established long-term clinical benefits, as well as potential changes in the cost structure of lifestyle-based interventions, including scalability, digital delivery, and implementation efficiency.

### Contribution to the Literature

This study contributes to the literature in four main ways. First, it provides an integrated comparison of dietary, pharmacological, and exercise-based interventions within the same older population, jointly considering metabolic outcomes, frailty, and economic value. Second, it shows that interventions with similar short-term clinical outcomes may differ meaningfully in cost-effectiveness and cost–utility depending on the evaluative framework applied. Third, it advances population health debates by linking intervention value to broader frameworks of healthy ageing, capability, and functioning. Fourth, the study demonstrates that economic conclusions are sensitive to both evaluative framework and pricing assumptions, highlighting the importance of integrating multiple perspectives in economic evaluation.

### Limitations

Several limitations should be acknowledged. First, the short follow-up period limits assessment of longer-term clinical, functional, and economic consequences, particularly for frailty trajectories and sustained quality-of-life change. Second, the relatively modest sample size may have limited power to detect small but meaningful between-group differences and has direct implications for the stability of the economic estimates. In particular, some responder-based ICERs were derived from very small numbers of observed events; for example, the exercise-arm responder rate was based on one responder among complete cases, making incremental effect estimates highly sensitive to small-sample variation and limiting the stability of ICER-based inference. Third, complete-case analysis may introduce bias if missingness was not random. Fourth, utility estimates were derived from mapped SF-12 data rather than direct preference-based measurement, which may add uncertainty. Although such mapping is common in economic evaluation when direct utility instruments are unavailable, it remains an indirect approach. Although the mapping approach has been validated in general populations, its performance in older adults with type 2 diabetes may introduce additional uncertainty, particularly when between-group differences in QALYs are small. In particular, regression-based mapping algorithms inevitably involve individual-level prediction error, and their performance may vary across clinical populations. Accordingly, the estimated QALYs should be interpreted as approximations of preference-based utility rather than direct utility measurements. In addition, no validated Israeli-specific mapping algorithm for deriving preference-based utility values from the SF-12 is currently available. Consequently, utility values were estimated using an internationally validated algorithm developed in the United States. Although this approach is widely accepted in trial-based economic evaluations, population-specific preference differences may influence estimated utility values. Future research should develop and validate Israeli-specific mapping algorithms to improve the precision of utility estimation in health economic evaluations conducted in Israel. The frailty phenotype was derived from available trial variables, with key objective components (grip strength and gait speed) based on direct measurement. The modified operationalization excluded the weight-loss and physical-activity components because intentional and unintentional weight loss could not be distinguished within the intervention setting, and physical activity represented a direct intervention target. While this approach was intended to reduce potential misclassification and improve internal validity, it also reduced direct comparability with the original Fried phenotype and may have reduced sensitivity to detecting intervention-related functional improvements. Consequently, some intervention-related improvements in frailty may have been conservatively estimated.

Fifth, the intervention cost perspective was deliberately narrow and did not capture broader healthcare utilization, informal care, patient time, travel costs, or downstream savings in hospitalization, disability, caregiving, or long-term care. As a result, the current cost estimates may underestimate the full economic burden or benefit of each intervention because indirect costs, patient time, intervention delivery time, and downstream healthcare consequences were not fully captured. It is important to note that the present analysis adopts a restricted intervention-cost perspective and does not capture broader societal costs and benefits. As such, the estimated economic value should be interpreted as a partial representation of overall value, which may differ under a full societal perspective. Under a broader healthcare-system or societal perspective, the relative economic value of the evaluated interventions could differ through the inclusion of downstream healthcare utilization, informal care, patient time, productivity, and long-term health consequences. Consequently, future studies adopting broader economic perspectives may provide a more comprehensive assessment of the comparative value of these interventions for healthcare decision-making.

Finally, trial conditions may not fully reflect real-world implementation, especially for lifestyle-based strategies whose effectiveness depends on adherence, context, and social support. Concepts such as physical resilience may also require longer follow-up windows and broader functional assessment than were possible in the present study [35, 36].

An additional limitation is that the primary composite endpoint may not have been equally proximal to all intervention mechanisms. Although the supplementary analyses partly address this concern, they remain exploratory and should not be interpreted as replacing the primary outcome framework.

In addition, the short duration of follow-up limits the ability to assess the sustainability of quality-of-life improvements and long-term economic value. Behavioral and lifestyle-related gains observed over short periods may attenuate over time, whereas pharmacological benefits may accumulate, particularly with respect to long-term complications. As a result, the present analysis may underestimate the long-term clinical and economic value of pharmacological treatment. Consequently, extrapolation of the present findings beyond the observed trial horizon should be avoided until confirmed by studies with longer follow-up and broader economic evaluations incorporating long-term outcomes.

## Conclusions

This study demonstrates that interventions with similar short-term clinical outcomes may differ substantially in their economic and population health value. In this sample of older adults, non-pharmacological interventions, particularly dietary strategies, were associated with markedly lower costs and more favorable utility-based outcomes within the short-term analytic horizon and under current pharmacological pricing conditions corresponding to patent-protected drug costs. Importantly, the observed advantage appears to be driven primarily by short-term differences in quality-of-life measures and should not be interpreted as indicating overall superiority over pharmacological treatment.

These findings should be interpreted within the short-term trial horizon and in the absence of long-term complication outcomes associated with pharmacological therapy. They should also be considered in light of the established long-term cardiovascular and renal benefits of SGLT2 inhibitors, as well as the likelihood of future reductions in pharmacological prices. More broadly, the results challenge the assumption within the context of short-term evaluation that the most clinically visible intervention is necessarily the most valuable from a societal perspective. In ageing populations, where policy goals increasingly include healthy ageing, functional ability, quality of life, and sustainability, value frameworks that move beyond biomedical endpoints are essential.

Overall, the results support cautious consideration of increased policy and research attention to preventive, community-based, and lifestyle-oriented models of care.

This conclusion is reinforced by scenario analyses, indicating that the economic value of such interventions remains relatively stable within the explored parameter ranges, even under plausible future reductions in pharmacological pricing. At the same time, these findings underscore that realizing the promise of such interventions requires attention to the social determinants and structural conditions that shape equitable access, adherence, and long-term effectiveness.

Responder-based cost-effectiveness results should be interpreted cautiously, given the modest sample and low event counts in some arms. Larger trials with longer follow-up and broader cost capture are needed to establish the long-term comparative value of these strategies with greater precision.

Future research should also consider broader endpoint frameworks that integrate metabolic control, function, perceived well-being, and treatment burden when evaluating interventions for older adults. Accordingly, the present findings should be interpreted as informing the complementary role of lifestyle interventions within integrated care strategies, rather than suggesting substitution of pharmacological therapy.

## Supplementary Information

Supplementary Material 1.

Supplementary Material 2.

Supplementary Material 3.

Supplementary Material 4.

Supplementary Material 5.

Supplementary Material 6.

Supplementary Material 7.

Supplementary Material 8.

Supplementary Material 9.

Supplementary Material 10.

Supplementary Material 11.

Supplementary Material 12.

Supplementary Material 13.

Supplementary Material 14.

## Data Availability

The datasets generated and/or analysed during the current study are not publicly available due to institutional restrictions but are available from the corresponding author on reasonable request.

## Declarations

### Use of artificial intelligence (AI) tools

AI-based language tools were used to assist with language editing and stylistic refinement, and AI-assisted coding tools were used to support Python-based implementation and figure generation. These tools were not involved in study design, data collection, statistical analysis, economic model development, interpretation of results, or the formulation of scientific conclusions. All statistical analyses and the economic model were designed and conducted by the authors. All AI-assisted content and code were critically reviewed, tested, and validated by the authors. Code execution and all reported results were verified through independent re-running by the authors. The authors take full responsibility for the integrity and accuracy of the work.

## Glossary

HbA1c: Hemoglobin A1c: Long-term glycemic control marker
SF-12: 12-Item Short Form Health Survey: Health-related quality-of-life questionnaire
PCS: Physical Component Summary: Physical health domain of SF-12
MCS: Mental Component Summary: Mental health domain of SF-12
BMI: Body Mass Index: Anthropometric measure of body composition
RMR: Resting Metabolic Rate: Energy expenditure at rest
QALY: Quality-Adjusted Life Year: Utility-based measure combining quality and quantity of life
SF-6D: Short Form 6 Dimensions: Preference-based utility measure derived from SF instruments
Δ (Delta): Change: Difference between time points
W0: Baseline (Week 0): Initial measurement point
W10: Week 10: Follow-up measurement point
VegMedD: Vegeterranean Mediterranean Diet: Plant-based Mediterranean dietary intervention
ICER: Incremental Cost-Effectiveness Ratio: Ratio of incremental cost to incremental effect
NMB: Net Monetary Benefit: Monetary valuation of intervention benefit
CEAC: Cost-Effectiveness Acceptability Curve: Probability of cost-effectiveness across thresholds
CE plane: Cost-Effectiveness Plane: Graphical representation of cost-effectiveness results
PSA: Probabilistic Sensitivity Analysis: Analysis incorporating parameter uncertainty
WTP: Willingness-To-Pay: Threshold value for decision-making
QALY PSA: QALY-based Probabilistic Sensitivity Analysis: PSA based on QALY outcomes
ILS: Israeli Shekels: Currency used in economic evaluation
ANCOVA: Analysis of Covariance: Regression model adjusting for baseline values
IPTW: Inverse Probability of Treatment Weighting: Propensity score-based weighting method
CI: Confidence Interval: Range of uncertainty around estimates
SD: Standard Deviation: Measure of variability
n: Sample Size: Number of observations
RCT: Randomized Controlled Trial: Experimental study design
S1–S14: Supplementary Tables: Additional results tables
A1–A3: Appendix Tables: Supporting methodological tables
