# Supplementary material for: Clinical similarity, economic divergence: cost-effectiveness and population health implications of dietary, pharmacological, and exercise interventions in older adults with type 2 diabetes: a secondary analysis of a randomized controlled trial
Source: BMC Geriatr. 2026 Jul 25;26:1321. doi: 10.1186/s12877-026-07994-7 (PMC13685177; doi:10.1186/s12877-026-07994-7)
Supplement: Supplementary file 1 [file 12877_2026_7994_MOESM4_ESM.docx]

**CONSORT 2010 Checklist**

**Adapted for a Secondary Analysis of a Previously Completed Randomized Controlled Trial**

**Manuscript Title:**

*Clinical similarity, economic divergence: Cost-effectiveness and population health implications of dietary, pharmacological, and exercise interventions in older adults with type 2 diabetes: a secondary analysis of a randomized controlled trial*

**Explanatory Note**

This manuscript reports a secondary economic analysis of data derived from the previously completed CEV-65 randomized controlled trial (ClinicalTrials.gov Identifier: **NCT03560375**). Reporting follows the CONSORT 2010 Statement where applicable to secondary analyses. Items relating to the conduct of the original randomized trial (including participant recruitment, randomization, allocation concealment, blinding, and participant flow before analysis) are reported in the original trial publication and are referenced accordingly.

| **Section/Topic** | **Item No.** | **Checklist item** | **Reported on page No.** |
| --- | --- | --- | --- |
| **Title and Abstract** | **1a** | Identification as a randomized trial in the title | **1** |
|  | **1b** | Structured summary of trial design, methods, results and conclusions | **2–3** |
| **Introduction** | **2a** | Scientific background and explanation of rationale | **4–5** |
|  | **2b** | Specific objectives or hypotheses | **5** |
| **Methods** | **3a** | Description of trial design | **5–6** |
|  | **3b** | Important changes to methods after trial commencement | **Not applicable** |
|  | **4a** | Eligibility criteria for participants | **6; additional details reported in the original trial publication** |
|  | **4b** | Settings and locations where the data were collected | **5–6; additional details reported in the original trial publication** |
|  | **5** | Interventions for each group | **5–6; detailed intervention protocol reported in the original trial publication** |
|  | **6a** | Completely defined prespecified outcome measures | **6–7** |
|  | **6b** | Changes to trial outcomes after trial commencement | **None** |
|  | **7a** | Sample size determination | **Reported in the original trial publication** |
|  | **7b** | Interim analyses and stopping guidelines | **Not applicable** |
|  | **8a** | Method used to generate the random allocation sequence | **Reported in the original trial publication** |
|  | **8b** | Type of randomisation | **Reported in the original trial publication** |
|  | **9** | Allocation concealment mechanism | **Reported in the original trial publication** |
|  | **10** | Implementation | **Reported in the original trial publication** |
|  | **11a** | Blinding | **Reported in the original trial publication** |
|  | **11b** | Similarity of interventions | **Reported in the original trial publication** |
|  | **12a** | Statistical methods used to compare groups | **7–9** |
|  | **12b** | Methods for additional analyses | **7–9** |
| **Results** | **13a** | Participant flow | **Supplementary CONSORT Flow Diagram; original trial publication** |
|  | **13b** | Losses and exclusions after randomisation | **Supplementary CONSORT Flow Diagram; original trial publication** |
|  | **14a** | Recruitment dates | **Reported in the original trial publication** |
|  | **14b** | Why the trial ended or was stopped | **Reported in the original trial publication** |
|  | **15** | Baseline demographic and clinical characteristics | **10 (Table 1)** |
|  | **16** | Numbers analysed | **10–14** |
|  | **17a** | Outcomes and estimation | **10–18** |
|  | **17b** | Presentation of binary outcomes | **11–12 (Table 2)** |
|  | **18** | Ancillary analyses | **7–18; Supplementary Material** |
|  | **19** | Harms | **Reported in the original trial publication; adverse events were not re-evaluated in the present secondary economic analysis** |
| **Discussion** | **20** | Trial limitations | **19–20** |
|  | **21** | Generalisability | **19–20** |
|  | **22** | Interpretation | **14–20** |
| **Other information** | **23** | Registration | **2; 5 (ClinicalTrials.gov Identifier: NCT03560375)** |
|  | **24** | Protocol | **Reported in the original trial publication** |
|  | **25** | Funding | **Declarations – Funding** |
