# Supplementary material for: Clinical similarity, economic divergence: cost-effectiveness and population health implications of dietary, pharmacological, and exercise interventions in older adults with type 2 diabetes: a secondary analysis of a randomized controlled trial
Source: BMC Geriatr. 2026 Jul 25;26:1321. doi: 10.1186/s12877-026-07994-7 (PMC13685177; doi:10.1186/s12877-026-07994-7)
Supplement: Supplementary file 2 [file 12877_2026_7994_MOESM6_ESM.docx]

**Supplementary Appendix**

**Appendix Methods**

**Data Preparation and Cleaning**

The analytic dataset was derived from the original study file:

**Data_CEV-65_12.11.2023_V68_AB_for_RMRs_paper_with_SB.xlsx**

The dataset contains multiple worksheets documenting participant characteristics, metabolic indicators, frailty assessments, physiological measures, and quality-of-life outcomes collected during the CEV-65 trial.

Data preparation involved the following steps:

1. Import of all relevant worksheets into the analytic environment.
2. Verification of participant identifiers across data sources.
3. Harmonization of variable names across worksheets.
4. Conversion of numeric variables stored as text to numeric format.
5. Validation of baseline and follow-up measurements.
6. Creation of derived variables required for the statistical analysis.

Derived variables included:

- change in HbA1c between baseline and week 10
- change in frailty score between baseline and week 10
- composite responder indicator for metabolic improvement
- mapped utility values derived from SF-12 health-related quality-of-life scores
- QALY estimates over the 10-week study horizon

Missing values were handled using **complete-case analysis for outcome-specific models**, consistent with the within-trial analytic framework.

**Appendix Statistical Models**

**ANCOVA Models**

Treatment effects on week-10 outcomes were estimated using analysis of covariance (ANCOVA) models adjusting for baseline values.

The general specification was:

$$Y_{i,10}=\beta_{0}+\beta_{1}{Treatment}_{i}+\beta_{2}Y_{i,0}+\beta_{3}{Age}_{i}+\beta_{4}{Sex}_{i}+\epsilon_{i}$$

where:

- $Y_{i,10}$ represents the week-10 outcome
- $Y_{i,0}$represents the baseline value of the outcome
- ${Treatment}_{i}$denotes the intervention group

Separate models were estimated for:

- HbA1c
- frailty score

These models correspond to the results reported in **Supplementary Table S1**

Frailty variables used in all models correspond to the operationalization described in the main text.

**Supplementary Table S1. ANCOVA Models for Week-10 Outcomes**

**HbA1c at week 10**

| **Term** | **Beta** | **95% CI** | **p-value** |
| --- | --- | --- | --- |
| Intercept | 3.2523 | 0.6364 to 5.8681 | 0.0155 |
| Exercise vs Empagliflozin | 0.1625 | -0.2047 to 0.5297 | 0.3809 |
| VegMedD vs Empagliflozin | -0.0753 | -0.4283 to 0.2778 | 0.6724 |
| Baseline HbA1c | 0.5924 | 0.4647 to 0.7202 | <0.001 |
| Age | -0.0108 | -0.0431 to 0.0216 | 0.5097 |
| Sex coded 1 | 0.1937 | -0.1056 to 0.4931 | 0.2014 |

**Frailty score at week 10**

| **Term** | **Beta** | **95% CI** | **p-value** |
| --- | --- | --- | --- |
| Intercept | -0.5095 | -3.2146 to 2.1956 | 0.7084 |
| Exercise vs Empagliflozin | 0.1376 | -0.2921 to 0.5673 | 0.5252 |
| VegMedD vs Empagliflozin | 0.2782 | -0.1299 to 0.6863 | 0.1783 |
| Baseline frailty score | 0.4731 | 0.2804 to 0.6658 | <0.001 |
| Age | 0.0078 | -0.0296 to 0.0452 | 0.6779 |
| Sex coded 1 | 0.3349 | -0.0185 to 0.6884 | 0.0629 |

**Caption**
Analysis of covariance models estimating adjusted between-group differences in week-10 HbA1c and Fried frailty score.

**Note**
Reference group = Empagliflozin. Each model was adjusted for the baseline value of the corresponding outcome, age, and sex. Frailty reflects the modified four-component operationalization excluding weight loss and physical activity.

**Mixed-Effects Model Specification**

Longitudinal changes in clinical outcomes were examined using linear mixed-effects models with participant-level random intercepts.

The model specification was:

$$Y_{i,t}=\beta_{0}+\beta_{1}{Treatment}_{i}+\beta_{2}{Time}_{i}+\beta_{3}({Treatment}_{i}\times{Time}_{i})+u_{i}+\epsilon_{it}$$

where:

- $Y_{i,t}$ denotes the outcome for participant (i) at time (t)
- ${Time}_{i}$ represents measurement time (baseline or week 10)
- $u_{i}$ represents the participant-level random intercept

These models correspond to the analyses reported in **Supplementary Table S2**.

Mixed-effects results are reported in **Table 4**. For HbA1c, there was a significant overall reduction over time (β for week 10 vs baseline = −0.4728, 95% CI −0.7625 to −0.1831, p = 0.0014). For frailty, the overall time effect approached statistical significance (β = −0.2485, 95% CI −0.4975 to 0.0005, p = 0.0505). None of the group-by-time interaction terms reached statistical significance.

**Supplementary Table S2. Mixed-Effects Models for Repeated Measures (W0 and W10)**

**HbA1c**

| **Term** | **Beta** | **95% CI** | **p-value** |
| --- | --- | --- | --- |
| Intercept | 9.6167 | 6.4868 to 12.7467 | <0.001 |
| Exercise vs Empagliflozin | -0.4171 | -0.9140 to 0.0797 | 0.0999 |
| VegMedD vs Empagliflozin | 0.0432 | -0.4667 to 0.5530 | 0.8682 |
| Week 10 vs baseline | -0.4728 | -0.7625 to -0.1831 | 0.0014 |
| Exercise × week 10 | 0.3271 | -0.0921 to 0.7463 | 0.1262 |
| VegMedD × week 10 | -0.1043 | -0.5141 to 0.3054 | 0.6177 |
| Age | -0.0276 | -0.0709 to 0.0157 | 0.2116 |
| Sex coded 1 | -0.1236 | -0.5116 to 0.2644 | 0.5323 |
| Group Var | 0.7313 | 0.1560 to 3.4265 | <0.001 |

**Frailty score**

| **Term** | **Beta** | **95% CI** | **p-value** |
| --- | --- | --- | --- |
| Intercept | 0.6776 | -1.8195 to 3.1747 | 0.5948 |
| Exercise vs Empagliflozin | -0.1474 | -0.5748 to 0.2799 | 0.4989 |
| VegMedD vs Empagliflozin | 0.1173 | -0.3198 to 0.5544 | 0.5990 |
| Week 10 vs baseline | -0.3618 | -0.6842 to -0.0394 | 0.0278 |
| Exercise × week 10 | 0.1944 | -0.2810 to 0.6698 | 0.4228 |
| VegMedD × week 10 | 0.1943 | -0.2583 to 0.6468 | 0.4001 |
| Age | 0.0038 | -0.0306 to 0.0382 | 0.8295 |
| Sex coded 1 | 0.3958 | 0.0757 to 0.7159 | 0.0154 |
| Group Var | 1.0716 | 0.6419 to 1.7889 | 0.0034 |

**Caption**
Mixed-effects models for repeated measures from baseline to week 10.

**Note**
Random intercepts were included at participant level. Reference group = Empagliflozin. Time was coded as week 10 vs baseline.

**Propensity Score Model**

To evaluate robustness of treatment comparisons, a multinomial propensity score model was estimated using multinomial logistic regression.

Covariates included:

- age
- sex
- body mass index (BMI)
- baseline HbA1c
- baseline frailty score
- resting metabolic rate
- systolic blood pressure
- diastolic blood pressure

Estimated propensity scores were used to construct **inverse probability of treatment weights (IPTW)**.

The weighted analyses correspond to **Supplementary Tables S3 and S4**.

The multinomial propensity score model is reported in **Supplementary Table S3**. Most baseline covariates were not significantly associated with treatment assignment. Diastolic blood pressure in the VegMedD versus Empagliflozin contrast reached nominal statistical significance, but the overall pattern remained consistent with limited baseline imbalance between groups.

**Supplementary Table S3. Multinomial Propensity Score Model**

**VegMedD vs Empagliflozin**

| **Variable** | **Beta** | **95% CI** | **p-value** |
| --- | --- | --- | --- |
| const | 0.1107 | -0.8871 to 1.1084 | 0.8279 |
| Age | -0.4664 | -1.0642 to 0.1314 | 0.1262 |
| Sex | -0.1977 | -1.5804 to 1.1851 | 0.7794 |
| BMI_W0 | -0.0014 | -0.6386 to 0.6358 | 0.9966 |
| HbA1c_W0 | -0.0684 | -0.5781 to 0.4414 | 0.7926 |
| Frailty4_W0 | 0.1052 | -0.4591 to 0.6695 | 0.7149 |
| RMR_W0 | 0.3175 | -0.5298 to 1.1649 | 0.4627 |
| Rest_BP_sys_AV_W0 | -0.0539 | -0.6784 to 0.5707 | 0.8657 |
| Rest_BP_dia_AV_W0 | 0.6186 | 0.0247 to 1.2125 | 0.0412 |

**Exercise vs Empagliflozin**

| **Variable** | **Beta** | **95% CI** | **p-value** |
| --- | --- | --- | --- |
| const | 0.2472 | -0.7266 to 1.2209 | 0.6188 |
| Age | -0.1574 | -0.6881 to 0.3733 | 0.5610 |
| Sex | -0.3131 | -1.6802 to 1.0541 | 0.6536 |
| BMI_W0 | 0.2447 | -0.3849 to 0.8743 | 0.4463 |
| HbA1c_W0 | -0.4680 | -1.0645 to 0.1286 | 0.1241 |
| Frailty4_W0 | -0.0746 | -0.6202 to 0.4709 | 0.7887 |
| RMR_W0 | 0.0131 | -0.7916 to 0.8177 | 0.9746 |
| Rest_BP_sys_AV_W0 | -0.4106 | -1.0405 to 0.2194 | 0.2015 |
| Rest_BP_dia_AV_W0 | 0.4791 | -0.1110 to 1.0691 | 0.1115 |

**Caption**
Multinomial logit propensity score model for intervention assignment.

**Note**
Empagliflozin was the reference group. Continuous covariates were standardized prior to model estimation, while sex was included as coded in the cleaned dataset. The model estimates the association between baseline characteristics and treatment assignment in a multinomial logit framework.

After IPTW, no weighted between-group differences reached statistical significance for either HbA1c or frailty at week 10 (**Supplementary Table S4**). The largest weighted contrast was Exercise versus Empagliflozin for HbA1c at week 10 (β = 0.4542, 95% CI −0.0355 to 0.9439, p = 0.0691), which remained non-significant.

**Supplementary Table S4. IPTW-Weighted Treatment Effects**

| **Outcome** | **Contrast** | **Beta** | **95% CI** | **p-value** |
| --- | --- | --- | --- | --- |
| HbA1c at week 10 | VegMedD vs Empagliflozin | 0.0240 | -0.4343 to 0.4824 | 0.9177 |
| HbA1c at week 10 | Exercise vs Empagliflozin | 0.1592 | -0.3268 to 0.6453 | 0.5190 |
| Frailty score at week 10 | VegMedD vs Empagliflozin | 0.2114 | -0.2690 to 0.6918 | 0.3817 |
| Frailty score at week 10 | Exercise vs Empagliflozin | -0.0277 | -0.5567 to 0.5012 | 0.9174 |

**Caption**
Inverse probability of treatment weighted estimates of week-10 treatment effects.

**Note**
Weighted linear regression models used stabilized IPTW from the multinomial propensity score model. Empagliflozin was the reference group.

**Appendix Economic Evaluation**

**Cost Inputs**

Intervention costs were estimated using official pharmaceutical price registries and published economic evaluations of dietary and exercise interventions.

Drug prices were obtained from the **Israeli Ministry of Health Drug Registry**, while dietary and exercise program costs were derived from published economic evaluations of Mediterranean diet interventions and supervised exercise programs.

All costs are reported in **Israeli Shekels (ILS)** and reflect the **2024 price year**.

Net monetary benefit estimates are shown in **Supplementary Table S5**. At λ = 5,000 ILS per responder, Empagliflozin yielded the highest NMB at λ = 5,000 ILS (872.6 ILS), followed by VegMedD (523.3 ILS) and Exercise (30.1 ILS).

**Supplementary Table S5. Net Monetary Benefit (NMB)**

| **Intervention** | **Mean cost (ILS)** | **Responder rate** | **NMB at λ=5,000 ILS** | **NMB at λ=10,000 ILS** |
| --- | --- | --- | --- | --- |
| Empagliflozin | 608.9 | 0.2963 | 872.6 | 2354.1 |
| Exercise | 197.2 | 0.0455 | 30.1 | 257.4 |
| VegMedD | 191.2 | 0.1429 | 523.3 | 1237.4 |

**Caption**
Net monetary benefit of each intervention under two willingness-to-pay thresholds.

**Note**
NMB = (λ × responder rate) − cost. Higher values indicate greater economic attractiveness. Responder rates are based on complete-case analysis using the revised frailty operationalization. Costs are presented in Israeli Shekels (ILS), 2024 price year.

**Utility and cost-utility analysis**

Utility and QALY results are presented in **Supplementary Table S6**. Mean utility increased from baseline to week 10 in the Exercise and VegMedD groups, whereas a slight decrease was observed in the Empagliflozin group. Mean QALYs over the 10-week horizon were 0.1481 in Empagliflozin, 0.1502 in Exercise, and 0.1536 in VegMedD.

**Supplementary Table S6. Utility and QALY Outcomes Derived from SF-12**

| **Intervention** | **n** | **Baseline utility mean ± SD** | **Week-10 utility mean ± SD** | **Change in utility mean ± SD** | **QALY mean ± SD** |
| --- | --- | --- | --- | --- | --- |
| Empagliflozin | 26 | 0.7769 ± 0.1005 | 0.7624 ± 0.1036 | -0.0146 ± 0.0946 | 0.1481 ± 0.0208 |
| Exercise | 19 | 0.7550 ± 0.1095 | 0.8067 ± 0.0891 | 0.0517 ± 0.0830 | 0.1502 ± 0.0200 |
| VegMedD | 26 | 0.7813 ± 0.0781 | 0.8158 ± 0.0693 | 0.0345 ± 0.0773 | 0.1536 ± 0.0154 |

**Caption**
Health utility and 10-week QALY estimates derived from paired SF-12 data, recalculated using patient-level observations.

**Note**
Utility values were calculated at the individual level using baseline and week-10 SF-12 physical and mental component scores, age, and sex.
Utility values were derived using a validated regression-based mapping approach from SF-12 to preference-based utility estimates.
QALYs were estimated using the area-under-the-curve approach over the 10-week study horizon.
Results are fully consistent with the probabilistic sensitivity analysis presented in Supplementary Table S11.

Cost-utility results are shown in **Supplementary Table S7**. Relative to Empagliflozin, both Exercise and VegMedD had lower costs and slightly higher QALYs, resulting in negative ICERs, which in this context indicate lower cost and greater effectiveness than the reference comparator.

**Supplementary Table S7. Cost-Utility Analysis**

| **Intervention** | **n** | **Mean cost (ILS)** | **Mean QALY** | **Incremental cost vs Empagliflozin** | **Incremental QALY vs Empagliflozin** | **ICER (ILS/QALY)** |
| --- | --- | --- | --- | --- | --- | --- |
| Empagliflozin | 26 | 608.9 | 0.1481 | 0.0 | 0.0000 | Reference |
| Exercise | 19 | 197.2 | 0.1502 | -411.7 | 0.0021 | -195226.40 |
| VegMedD | 26 | 191.2 | 0.1536 | -417.7 | 0.0055 | -75853.22 |

**Caption**
Cost-utility analysis comparing each intervention with Empagliflozin as the reference strategy.

**Note**
QALYs were derived from patient-level SF-12 data using the same mapping and calculation procedures applied in Supplementary Table S6 and Supplementary Table S11. Negative ICER values indicate dominance (lower cost and higher QALYs).

ICER = incremental cost-effectiveness ratio; QALY = quality-adjusted life year. Costs are presented in Israeli Shekels (ILS), 2024 price year.

These results are consistent with the patient-level probabilistic sensitivity analysis reported in Supplementary Table S11.

**Supplementary Table S8. SF-12 Physical and Mental Component Scores**

| **Outcome** | **VegMedD** | **Empagliflozin** | **Exercise** | **p-value** |
| --- | --- | --- | --- | --- |
| Physical component score at week 10 | 45.55 ± 8.84 (29) | 43.61 ± 11.97 (26) | 44.89 ± 9.84 (23) | 0.7800 |
| Change in physical component score (W10−W0) | 0.13 ± 8.28 (27) | 1.33 ± 10.96 (26) | 3.56 ± 6.91 (20) | 0.4385 |
| Mental component score at week 10 | 53.00 ± 9.33 (28) | 49.37 ± 11.44 (26) | 53.34 ± 9.87 (23) | 0.3126 |
| Change in mental component score (W10−W0) | 4.25 ± 10.22 (26) | -3.08 ± 11.02 (26) | 2.47 ± 11.25 (19) | 0.0466 |

**Caption**

Comparison of physical and mental health-related quality-of-life outcomes across VegMedD, Empagliflozin, and Exercise groups, including change from baseline to week 10.

**Note**

- Values are mean ± standard deviation (n).
- Change scores are calculated as week 10 minus baseline.
- Positive values indicate improvement.
- p-values are derived from one-way ANOVA.
- MCS contributes directly to utility estimation and QALY calculation.

**Supplementary Table S9. Exercise-sensitive functional outcomes by intervention group**

| **Outcome** | **VegMedD** | **Empagliflozin** | **Exercise** | **p-value** |
| --- | --- | --- | --- | --- |
| Timed Up and Go at week 10, s | 10.14 ± 1.61 (29) | 10.75 ± 2.15 (29) | 10.16 ± 2.02 (25) | 0.4048 |
| Change in Timed Up and Go (W10−W0), s | -0.45 ± 1.11 (29) | -0.04 ± 1.11 (29) | -0.50 ± 1.09 (25) | 0.2477 |
| 30-s sit-to-stand at week 10, repetitions | 10.75 ± 1.84 (28) | 10.65 ± 3.37 (26) | 12.36 ± 2.13 (22) | 0.0404 |
| Change in 30-s sit-to-stand (W10−W0), repetitions | -0.14 ± 1.69 (28) | -0.38 ± 1.53 (26) | 0.95 ± 1.53 (22) | 0.0119 |
| 2-min walk at week 10, m | 165.40 ± 25.57 (28) | 162.36 ± 38.03 (26) | 167.55 ± 22.28 (22) | 0.8302 |
| Change in 2-min walk (W10−W0), m | 3.23 ± 18.56 (28) | -1.50 ± 14.69 (26) | 2.23 ± 10.62 (22) | 0.5004 |
| Skeletal muscle mass at week 10, kg | 27.61 ± 6.31 (29) | 25.68 ± 5.14 (29) | 28.00 ± 5.55 (25) | 0.2708 |
| Change in skeletal muscle mass (W10−W0), kg | -0.05 ± 0.67 (29) | -0.11 ± 0.80 (29) | -0.00 ± 0.69 (25) | 0.8544 |

**Caption**

Week-10 values and changes from baseline for outcomes expected to be particularly relevant to the exercise intervention.

**Note**

Values are mean ± standard deviation (n). p-values are from one-way ANOVA across the three intervention groups. For Timed Up and Go, negative change values indicate improvement. For sit-to-stand, 2-min walk, and skeletal muscle mass, positive change values indicate improvement.

Statistically significant differences were observed for 30-s sit-to-stand performance, both at week 10 and in change from baseline, indicating greater functional improvement in the exercise group.

**Supplementary Table S10. Additional ANCOVA models for reviewer-requested outcomes**

| **Outcome** | **Term** | **Beta** | **95% CI** | **p-value** |
| --- | --- | --- | --- | --- |
| Mental component score at week 10 | Intercept | 26.6166 | -8.9955 to 62.2288 | 0.1404 |
| Mental component score at week 10 | Exercise vs Empagliflozin | 4.7079 | -0.8920 to 10.3079 | 0.0980 |
| Mental component score at week 10 | VegMedD vs Empagliflozin | 5.6558 | 0.4502 to 10.8614 | 0.0337 |
| 30-s sit-to-stand at week 10 | Intercept | 4.1747 | -2.0424 to 10.3918 | 0.1848 |
| 30-s sit-to-stand at week 10 | Exercise vs Empagliflozin | 1.3778 | 0.4740 to 2.2816 | 0.0033 |
| 30-s sit-to-stand at week 10 | VegMedD vs Empagliflozin | 0.1596 | -0.6896 to 1.0089 | 0.7089 |

**Caption**
Adjusted between-group differences for week-10 mental component score and 30-s sit-to-stand performance.

**Note**
Empagliflozin was the reference group. Models were adjusted for the baseline value of the corresponding outcome, age, and sex. Beta coefficients represent adjusted mean differences at week 10 relative to Empagliflozin.
Results indicate a statistically significant improvement in mental component score for VegMedD and in functional performance (30-s sit-to-stand) for the exercise intervention, supporting the interpretation that different intervention modalities may affect distinct dimensions of health-related quality of life and function.

**Supplementary Table S11. Probabilistic Sensitivity Analysis for Cost-Utility Outcomes**

| **Comparison** | **Mean incremental cost (ILS)** | **Mean incremental QALY** | **Probability CE at λ = 0 ILS/QALY** | **Probability CE at λ = 50,000 ILS/QALY** | **Probability CE at λ = 100,000 ILS/QALY** | **Probability CE at λ = 150,000 ILS/QALY** | **Probability CE at λ = 200,000 ILS/QALY** | **Probability CE at λ = 300,000 ILS/QALY** |
| --- | --- | --- | --- | --- | --- | --- | --- | --- |
| VegMedD vs Empagliflozin | -421.40 | 0.00529 | 1.0000 | 0.9930 | 0.9710 | 0.9510 | 0.9390 | 0.9240 |
| Exercise vs Empagliflozin | -417.38 | 0.00196 | 0.9990 | 0.9310 | 0.8380 | 0.7680 | 0.7420 | 0.7020 |

**Caption**
Probability that VegMedD and Exercise are cost-effective relative to Empagliflozin across increasing willingness-to-pay thresholds per QALY, based on bootstrap resampling of patient-level QALYs and probabilistic cost simulation.

**Note**
QALYs were estimated at the individual level using baseline and week-10 SF-12 physical and mental component scores, with utilities derived via a validated mapping approach and aggregated over time using an area-under-the-curve method for the 10-week study horizon. Non-parametric bootstrap resampling (1,000 iterations) was applied within each intervention arm to jointly estimate incremental costs and QALYs. Intervention costs were modeled using Gamma distributions parameterized by base-case means and standard deviations (Appendix Table A2), while uncertainty in effectiveness was captured through resampling of patient-level data. Cost-effectiveness probabilities were calculated as the proportion of bootstrap iterations with positive incremental net monetary benefit. CE = cost-effective; λ = willingness-to-pay threshold per QALY; ILS = Israeli Shekels. Results correspond directly to the cost-utility acceptability curve presented in Supplementary Figure S8 and are based on the same patient-level QALY data reported in Supplementary Table S6, ensuring full consistency with the deterministic cost–utility results presented in Supplementary Table S7.

**Supplementary Table S12. One-way sensitivity analysis for empagliflozin price**

| **Empagliflozin cost (ILS, 10-week)** | **NMB Empagliflozin (λ=5,000)** | **NMB VegMedD** | **NMB Exercise** | **NMB Empagliflozin (λ=100,000/QALY)** | **NMB VegMedD** | **NMB Exercise** |
| --- | --- | --- | --- | --- | --- | --- |
| 608.9 (base case) | 872.6 | 523.3 | 30.1 | 14,201.1 | 15,168.8 | 14,822.8 |
| 456.7 (−25%) | 1,024.8 | 523.3 | 30.1 | 14,353.3 | 15,168.8 | 14,822.8 |
| 304.5 (−50%) | 1,177.0 | 523.3 | 30.1 | 14,505.5 | 15,168.8 | 14,822.8 |
| 152.2 (−75%) | 1,329.3 | 523.3 | 30.1 | 14,657.8 | 15,168.8 | 14,822.8 |
| 289.75 (SGLT2 inhibitor class-equivalent scenario) | 1,191.7 | 523.3 | 30.1 | 14,520.3 | 15,168.8 | 14,822.8 |

**Caption**
One-way sensitivity analysis examining the impact of lower empagliflozin prices on net monetary benefit.

**Note**
Responder-based NMB was calculated at λ = 5,000 ILS per responder, and QALY-based NMB was calculated at λ = 100,000 ILS per QALY. Empagliflozin costs were varied to reflect alternative pricing scenarios, including a class-equivalent SGLT2 inhibitor scenario based on lower-cost alternatives (e.g., dapagliflozin). Clinical outcomes and QALYs were assumed to remain unchanged across scenarios. In scenario analyses, the pharmacological arm is interpreted as representing the broader SGLT2 inhibitor class under alternative pricing assumptions.

**Supplementary Table S13. Threshold analysis for empagliflozin cost**

| **Comparison** | **WTP threshold** | **Threshold cost (ILS, 10-week)** |
| --- | --- | --- |
| vs VegMedD | 5,000 | 958.40 |
| vs Exercise | 5,000 | 1,451.41 |
| vs VegMedD | 50,000/QALY | Negative (no crossover) |
| vs Exercise | 100,000/QALY | Negative (no crossover) |

**Caption**
Threshold analysis identifying empagliflozin costs at which net monetary benefit equals that of comparator interventions.

**Note**
Negative thresholds indicate that no positive pharmacological cost would reverse the ranking under the specified willingness-to-pay threshold.
Threshold values represent the maximum empagliflozin cost at which its net monetary benefit remains equal to or greater than the comparator at the specified willingness-to-pay threshold.

**Supplementary Table S14. Class-equivalent SGLT2 scenario**

| **Intervention** | **Cost (ILS)** | **Incremental cost** | **Incremental effect** | **ICER (ILS per responder)** |
| --- | --- | --- | --- | --- |
| SGLT2 inhibitor (class-equivalent scenario) | 289.75 | 0 | 0 | Reference |
| VegMedD | 191.2 | −98.55 | −0.1534 | 642.30 |
| Exercise | 197.2 | −92.55 | −0.2508 | 368.97 |

**Caption**
Cost-effectiveness results under a class-equivalent SGLT2 inhibitor pricing scenario.

**Note**
The pharmacological cost was replaced with a lower-cost alternative based on class-equivalent SGLT2 inhibitor pricing (e.g., dapagliflozin). Clinical outcomes and QALYs were assumed to remain unchanged across scenarios.
The SGLT2 inhibitor arm is used as the reference comparator; therefore, its incremental cost and incremental effect are defined as zero by construction. Because both incremental costs and effects are negative, ICER values should be interpreted as the cost per responder lost when moving from the reference comparator to the alternative strategy.

**Appendix Table A1**

**Cost Inputs and Assumptions Used in the Economic Evaluation**

| **Cost Component** | **Unit Cost (ILS)** | **Unit** | **Assumed Quantity per Participant** | **Total Cost (ILS)** | **Data Source** |
| --- | --- | --- | --- | --- | --- |
| Empagliflozin pharmacotherapy | 60.9 | per week | 10 weeks | 608.9 | Israeli Ministry of Health Drug Registry |
| Mediterranean diet intervention (VegMedD) | 95.6 | per consultation | 2 sessions | 191.2 | Mediterranean diet intervention economic studies |
| Supervised exercise intervention | 98.6 | per program period | 2 sessions | 197.2 | Community exercise program economic evaluations |

**Notes**

- Costs represent **direct intervention costs** included in the base-case economic evaluation.
- All costs are expressed in **Israeli Shekels (ILS)**.
- When necessary, values reported in international studies were converted to ILS using contemporaneous exchange rates.
- Sensitivity analyses varied intervention cost parameters by **±20%**.

**Sources and Links**

**Pharmaceutical Pricing**

**Israeli Ministry of Health Drug Registry**
[**https://israeldrugs.health.gov.il**](https://israeldrugs.health.gov.il)

**DrugBank – Empagliflozin pharmacology and pricing context**
[**https://go.drugbank.com/drugs/DB09038**](https://go.drugbank.com/drugs/DB09038)

**Dietary Intervention Economic Evidence**

**Mediterranean Diet Cardiovascular Prevention Trial**
[**https://www.nejm.org/doi/full/10.1056/NEJMoa1800389**](https://www.nejm.org/doi/full/10.1056/NEJMoa1800389)

**Lifestyle intervention cost analysis**
[**https://pubmed.ncbi.nlm.nih.gov/30879178/**](https://pubmed.ncbi.nlm.nih.gov/30879178/)

**Exercise Intervention Economic Studies**

**Economic evaluation of supervised exercise programs
https://pubmed.ncbi.nlm.nih.gov/26711241/**

**Cost analysis of community physical activity interventions**
[**https://pubmed.ncbi.nlm.nih.gov/30533552/**](https://pubmed.ncbi.nlm.nih.gov/30533552/)

**Appendix Table A2**

**Parameter Distributions Used in Probabilistic Sensitivity Analysis**

| **Parameter** | **Base-case value** | **Distribution** | **Parameterization** | **Rationale** |
| --- | --- | --- | --- | --- |
| Empagliflozin cost | 608.9 | Gamma | mean=608.9, SD=121.8 | Positive cost distribution |
| VegMedD cost | 191.2 | Gamma | mean=191.2, SD=38.2 | Cost uncertainty |
| Exercise cost | 197.2 | Gamma | mean=197.2, SD=39.4 | Cost uncertainty |
| Empagliflozin responder probability | 0.2963 | Beta | α=8, β=19 | Observed responders |
| VegMedD responder probability | 0.1429 | Beta | α=4, β=24 | Observed responders |
| Exercise responder probability | 0.0455 | Beta | α=1, β=21 | Observed responders |
| Utility parameters | observed | Beta | estimated from mean and variance | Utilities bounded 0–1 |

**Notes**

Gamma distributions were assigned to **cost parameters**, reflecting non-negative right-skewed distributions typically observed in economic data.

Beta distributions were assigned to **probabilities and utilities**, reflecting values bounded between 0 and 1.

Beta distribution parameters were defined as α = number of responders and β = number of non-responders, ensuring that the distribution mean corresponds exactly to the observed base-case responder rate.

**Appendix Table A3**

**Bootstrap Cost-Effectiveness Results**

| **Comparison** | **Mean Incremental Cost (ILS)** | **Mean Incremental Effect** | **ICER (ILS per responder)** | **Probability CE λ=1,000** | **Probability CE λ=5,000** | **Probability CE λ=10,000** |
| --- | --- | --- | --- | --- | --- | --- |
| VegMedD vs Empagliflozin | -417.7 | -0.1534 | 2722.25 | 0.992 | 0.320 | 0.137 |
| Exercise vs Empagliflozin | -411.7 | -0.2508 | 1641.27 | 0.948 | 0.039 | 0.021 |

**Notes**

Results are based on 1,000 non-parametric bootstrap simulations resampling participants from the analytic cohort.

Incremental costs and effects were calculated relative to Empagliflozin, which served as the reference intervention.

ICER values may differ slightly from the base-case estimates reported in Table 3 due to bootstrap resampling variability.

Probabilities of cost-effectiveness are based on the proportion of bootstrap replications with positive incremental net monetary benefit (NMB) at each willingness-to-pay threshold.

The ICER is defined as:

$$ICER=\frac{\Delta C}{\Delta E}$$

where ΔC denotes incremental cost and ΔE denotes incremental effectiveness

The probability of cost-effectiveness corresponds to the proportion of bootstrap simulations in which the intervention produced a **positive net monetary benefit (NMB)** at the specified willingness-to-pay threshold.

These results correspond to the graphical results shown in:

- **Figure 1 – Cost-effectiveness plane**
- **Figure 2 – Cost-effectiveness acceptability curve**

**Economic Uncertainty Analysis**

**Cost-Effectiveness Plane**

The cost-effectiveness plane plots the bootstrap replications of incremental costs and effects.

Each point represents one bootstrap sample.

Interpretation:

- **Southeast quadrant:** lower cost and greater effectiveness
- **Northwest quadrant:** higher cost and lower effectiveness

These results are presented in **Figure 1 of the main manuscript**.

**Cost-Effectiveness Acceptability Curve**

The cost-effectiveness acceptability curve summarizes the probability that each intervention is economically preferred across willingness-to-pay thresholds ranging from **0 to 10,000 ILS**.

The CEAC is presented in **Figure 2**.

**Supplementary Figures**

The supplementary figures provide additional graphical analyses supporting the main findings, including participant flow, clinical outcome trajectories, propensity score diagnostics, covariate balance assessment, and deterministic sensitivity analyses.

**Supplementary Figure CONSORT. Flow diagram for the present secondary economic analysis**

**Caption**

CONSORT flow diagram summarizing participant allocation and the outcome-specific complete-case samples included in the present secondary economic evaluation.

**Note**

This manuscript reports a secondary economic analysis of the previously completed CEV-65 randomized controlled trial (ClinicalTrials.gov Identifier: NCT03560375). Participant screening, exclusions before randomization, allocation procedures, allocation concealment, blinding, recruitment, and follow-up are reported in the original trial publication. The present diagram summarizes participant allocation and the outcome-specific complete-case samples included in the current economic analyses. Outcome-specific complete-case sample sizes differ because each endpoint was analyzed using all participants with available data for that specific outcome. Consequently, the numbers included in the HbA1c, frailty, and composite responder analyses are not identical. The present flow diagram is intended to summarize the analytic samples used in this secondary economic evaluation rather than to reproduce the complete participant flow of the original randomized controlled trial.

**Supplementary Figure S1. Change in HbA1c by intervention group**

**Caption**

Distribution of individual changes in HbA1c from baseline to week 10 according to intervention group.

**Note**

Negative values indicate improvement in glycemic control. Boxes represent the interquartile range, horizontal lines indicate the median, whiskers represent 1.5 × the interquartile range, and individual observations are displayed.

**Abbreviations:** HbA1c = glycated hemoglobin.

**Supplementary Figure S2. Change in Fried frailty score by intervention group**

**Caption**

Distribution of individual changes in Fried frailty score from baseline to week 10 according to intervention group.

**Note**

Negative values indicate improvement in frailty status. The figure illustrates the distribution, central tendency, and variability of changes across intervention groups.

**Supplementary Figure S3. Propensity score overlap across intervention groups**

**Caption**

Distribution of estimated propensity scores across intervention groups before inverse probability of treatment weighting.

**Note**

The figure shows the distribution of estimated propensity scores across the observed treatment groups. Adequate overlap supports comparability between groups and the validity of inverse probability weighting analyses.

**Abbreviations:** IPTW = inverse probability of treatment weighting.

**Supplementary Figure S4. Covariate balance before and after inverse probability of treatment weighting**

**Caption**

Covariate balance before and after inverse probability of treatment weighting based on the multinomial propensity score model.

**Note**

Standardized mean differences are presented for each covariate before and after weighting. Covariates include age, sex, body mass index, baseline HbA1c, Fried frailty score, resting metabolic rate, systolic blood pressure, and diastolic blood pressure. Values closer to zero indicate improved balance across intervention groups. The vertical reference line represents a standardized mean difference of 0.10, commonly used as the threshold for acceptable covariate balance.

**Abbreviations:** IPTW = inverse probability of treatment weighting.

**Supplementary Figure S5. Tornado diagram of deterministic sensitivity analysis**

**Caption**

Deterministic sensitivity analysis (tornado diagram) showing the impact of individual parameter variation on incremental net monetary benefit (INMB).

**Note**

Each bar represents the change in incremental net monetary benefit when a single model parameter is varied across its plausible range while all remaining parameters are held constant. Results are presented for the VegMedD versus Empagliflozin comparison at a willingness-to-pay threshold of λ = 5,000 ILS per composite responder. The vertical reference line represents the base-case incremental net monetary benefit. Parameters are ordered according to their relative influence on model outcomes.

**Abbreviations:** INMB = incremental net monetary benefit; ILS = Israeli Shekels.

**Supplementary Figure S6. Change in SF-12 Mental Component Summary (MCS) score by intervention group**

**Caption**

Distribution of individual changes in SF-12 Mental Component Summary (MCS) score from baseline to week 10 according to intervention group.

**Note**

Higher values indicate improvement in perceived mental well-being. This figure complements Supplementary Table S8 and supports interpretation of the health-related quality-of-life differences incorporated into the cost–utility analysis.

**Abbreviations:** MCS = Mental Component Summary; SF-12 = 12-Item Short Form Health Survey.

**Supplementary Figure S7. Change in 30-second sit-to-stand performance by intervention group**

**Caption**

Distribution of individual changes in 30-second sit-to-stand performance from baseline to week 10 according to intervention group.

**Note**

Higher values indicate improvement in lower-extremity functional performance. This figure complements Supplementary Table S9 and illustrates the functionally proximal benefits observed in the exercise intervention group.

**Supplementary Figure S8. Cost–utility acceptability curve based on patient-level QALY estimates**

**Caption**

Cost–utility acceptability curve showing the probability that VegMedD and Exercise are cost-effective relative to Empagliflozin across willingness-to-pay thresholds based on QALY outcomes.

**Note**

Probabilities were estimated using non-parametric bootstrap resampling of patient-level QALY estimates combined with Gamma-distributed intervention costs. Results correspond directly to Supplementary Table S11 and are derived from the same patient-level bootstrap simulations.

**Abbreviations:** CEAC = cost-effectiveness acceptability curve; QALY = quality-adjusted life year.

**Supplementary Figure S9. One-way sensitivity analysis of responder-based net monetary benefit**

**Caption**

One-way deterministic sensitivity analysis of responder-based net monetary benefit across alternative empagliflozin price scenarios.

**Note**

Lines represent net monetary benefit calculated at a willingness-to-pay threshold of λ = 5,000 ILS per composite responder. The analysis illustrates the robustness of the economic conclusions across plausible variations in empagliflozin acquisition costs.

**Abbreviations:** NMB = net monetary benefit; ILS = Israeli Shekels.

**Supplementary Figure S10. One-way sensitivity analysis of QALY-based net monetary benefit**

**Caption**

One-way deterministic sensitivity analysis of QALY-based net monetary benefit across alternative empagliflozin price scenarios.

**Note**

Lines represent net monetary benefit calculated at a willingness-to-pay threshold of λ = 100,000 ILS per QALY. The analysis demonstrates the robustness of the cost–utility findings across plausible variations in empagliflozin acquisition costs.

**Abbreviations:** NMB = net monetary benefit; QALY = quality-adjusted life year; ILS = Israeli Shekels.
