# Supplementary material for: Clinical similarity, economic divergence: cost-effectiveness and population health implications of dietary, pharmacological, and exercise interventions in older adults with type 2 diabetes: a secondary analysis of a randomized controlled trial
Source: BMC Geriatr. 2026 Jul 25;26:1321. doi: 10.1186/s12877-026-07994-7 (PMC13685177; doi:10.1186/s12877-026-07994-7)
Supplement: Supplementary file 3 [file 12877_2026_7994_MOESM7_ESM.docx]

**Supplementary Figures**

The supplementary figures provide additional graphical analyses supporting the main findings, including participant flow, clinical outcome trajectories, propensity score diagnostics, covariate balance assessment, and deterministic sensitivity analyses.

**Supplementary Figure CONSORT. Flow diagram for the present secondary economic analysis**

**Caption**

CONSORT flow diagram summarizing participant allocation and the outcome-specific complete-case samples included in the present secondary economic evaluation.

**Note**

This manuscript reports a secondary economic analysis of the previously completed CEV-65 randomized controlled trial (ClinicalTrials.gov Identifier: NCT03560375). Participant screening, exclusions before randomization, allocation procedures, allocation concealment, blinding, recruitment, and follow-up are reported in the original trial publication. The present diagram summarizes participant allocation and the outcome-specific complete-case samples included in the current economic analyses. Outcome-specific complete-case sample sizes differ because each endpoint was analyzed using all participants with available data for that specific outcome. Consequently, the numbers included in the HbA1c, frailty, and composite responder analyses are not identical. The present flow diagram is intended to summarize the analytic samples used in this secondary economic evaluation rather than to reproduce the complete participant flow of the original randomized controlled trial.

**Supplementary Figure S1. Change in HbA1c by intervention group**

**Caption**

Distribution of individual changes in HbA1c from baseline to week 10 according to intervention group.

**Note**

Negative values indicate improvement in glycemic control. Boxes represent the interquartile range, horizontal lines indicate the median, whiskers represent 1.5 × the interquartile range, and individual observations are displayed.

**Abbreviations:** HbA1c = glycated hemoglobin.

**Supplementary Figure S2. Change in Fried frailty score by intervention group**

**Caption**

Distribution of individual changes in Fried frailty score from baseline to week 10 according to intervention group.

**Note**

Negative values indicate improvement in frailty status. The figure illustrates the distribution, central tendency, and variability of changes across intervention groups.

**Supplementary Figure S3. Propensity score overlap across intervention groups**

**Caption**

Distribution of estimated propensity scores across intervention groups before inverse probability of treatment weighting.

**Note**

The figure shows the distribution of estimated propensity scores across the observed treatment groups. Adequate overlap supports comparability between groups and the validity of inverse probability weighting analyses.

**Abbreviations:** IPTW = inverse probability of treatment weighting.

**Supplementary Figure S4. Covariate balance before and after inverse probability of treatment weighting**

**Caption**

Covariate balance before and after inverse probability of treatment weighting based on the multinomial propensity score model.

**Note**

Standardized mean differences are presented for each covariate before and after weighting. Covariates include age, sex, body mass index, baseline HbA1c, Fried frailty score, resting metabolic rate, systolic blood pressure, and diastolic blood pressure. Values closer to zero indicate improved balance across intervention groups. The vertical reference line represents a standardized mean difference of 0.10, commonly used as the threshold for acceptable covariate balance.

**Abbreviations:** IPTW = inverse probability of treatment weighting.

**Supplementary Figure S5. Tornado diagram of deterministic sensitivity analysis**

**Caption**

Deterministic sensitivity analysis (tornado diagram) showing the impact of individual parameter variation on incremental net monetary benefit (INMB).

**Note**

Each bar represents the change in incremental net monetary benefit when a single model parameter is varied across its plausible range while all remaining parameters are held constant. Results are presented for the VegMedD versus Empagliflozin comparison at a willingness-to-pay threshold of λ = 5,000 ILS per composite responder. The vertical reference line represents the base-case incremental net monetary benefit. Parameters are ordered according to their relative influence on model outcomes.

**Abbreviations:** INMB = incremental net monetary benefit; ILS = Israeli Shekels.

**Supplementary Figure S6. Change in SF-12 Mental Component Summary (MCS) score by intervention group**

**Caption**

Distribution of individual changes in SF-12 Mental Component Summary (MCS) score from baseline to week 10 according to intervention group.

**Note**

Higher values indicate improvement in perceived mental well-being. This figure complements Supplementary Table S8 and supports interpretation of the health-related quality-of-life differences incorporated into the cost–utility analysis.

**Abbreviations:** MCS = Mental Component Summary; SF-12 = 12-Item Short Form Health Survey.

**Supplementary Figure S7. Change in 30-second sit-to-stand performance by intervention group**

**Caption**

Distribution of individual changes in 30-second sit-to-stand performance from baseline to week 10 according to intervention group.

**Note**

Higher values indicate improvement in lower-extremity functional performance. This figure complements Supplementary Table S9 and illustrates the functionally proximal benefits observed in the exercise intervention group.

**Supplementary Figure S8. Cost–utility acceptability curve based on patient-level QALY estimates**

**Caption**

Cost–utility acceptability curve showing the probability that VegMedD and Exercise are cost-effective relative to Empagliflozin across willingness-to-pay thresholds based on QALY outcomes.

**Note**

Probabilities were estimated using non-parametric bootstrap resampling of patient-level QALY estimates combined with Gamma-distributed intervention costs. Results correspond directly to Supplementary Table S11 and are derived from the same patient-level bootstrap simulations.

**Abbreviations:** CEAC = cost-effectiveness acceptability curve; QALY = quality-adjusted life year.

**Supplementary Figure S9. One-way sensitivity analysis of responder-based net monetary benefit**

**Caption**

One-way deterministic sensitivity analysis of responder-based net monetary benefit across alternative empagliflozin price scenarios.

**Note**

Lines represent net monetary benefit calculated at a willingness-to-pay threshold of λ = 5,000 ILS per composite responder. The analysis illustrates the robustness of the economic conclusions across plausible variations in empagliflozin acquisition costs.

**Abbreviations:** NMB = net monetary benefit; ILS = Israeli Shekels.

**Supplementary Figure S10. One-way sensitivity analysis of QALY-based net monetary benefit**

**Caption**

One-way deterministic sensitivity analysis of QALY-based net monetary benefit across alternative empagliflozin price scenarios.

**Note**

Lines represent net monetary benefit calculated at a willingness-to-pay threshold of λ = 100,000 ILS per QALY. The analysis demonstrates the robustness of the cost–utility findings across plausible variations in empagliflozin acquisition costs.

**Abbreviations:** NMB = net monetary benefit; QALY = quality-adjusted life year; ILS = Israeli Shekels.
